# Skin cell-derived extracellular vesicles: a promising therapeutic strategy for cutaneous injury

**DOI:** 10.1093/burnst/tkac037

**Published:** 2022-10-18

**Authors:** Min Wang, Peipei Wu, Jin Huang, Wenhui Liu, Hui Qian, Yaoxiang Sun, Hui Shi

**Affiliations:** Jiangsu Key Laboratory of Medical Science and Laboratory Medicine, Institute of Stem Cell, School of Medicine, Jiangsu University, Zhenjiang 212000, China; Department of Clinical Laboratory, The Affiliated Yixing Hospital of Jiangsu University, Yixing 214200, China; Department of Clinical Laboratory, The Affiliated Yixing Hospital of Jiangsu University, Yixing 214200, China; Jiangsu Key Laboratory of Medical Science and Laboratory Medicine, Institute of Stem Cell, School of Medicine, Jiangsu University, Zhenjiang 212000, China; Jiangsu Key Laboratory of Medical Science and Laboratory Medicine, Institute of Stem Cell, School of Medicine, Jiangsu University, Zhenjiang 212000, China; Jiangsu Key Laboratory of Medical Science and Laboratory Medicine, Institute of Stem Cell, School of Medicine, Jiangsu University, Zhenjiang 212000, China; Department of Clinical Laboratory, The Affiliated Yixing Hospital of Jiangsu University, Yixing 214200, China; Jiangsu Key Laboratory of Medical Science and Laboratory Medicine, Institute of Stem Cell, School of Medicine, Jiangsu University, Zhenjiang 212000, China; Aoyang Institute of Cancer, Affiliated Aoyang Hospital of Jiangsu University, 279 Jingang Road, Suzhou, Jiangsu 215100, China

**Keywords:** Extracellular vesicles, Specific skin tissue cell, Cutaneous injury, Wound healing

## Abstract

Wound healing refers to the healing process that occurs after the skin and other tissues are separated or damaged by internal or external forces. It is a complex combination of tissue regeneration, granulation tissue hyperplasia, and scar formation, and shows the synergistic effects of these processes. After skin damage, the environment around the wound and the cells at site of the damage respond immediately, and a range of cytokines and growth factors are released. In cutaneous injury, extracellular vesicle (EV) signaling plays a vital role in the healing process via paracrine and endocrine mechanisms. EVs are natural intercellular and inter-organ communication tools that carry various bioactive substances for message exchange. Stem cells and stem cell EVs facilitate tissue repair, showing promising potential in regenerative medicine. Nevertheless, EVs derived from specific skin tissue cells, such as epidermal cells, fibroblasts, vascular endothelial cells and inflammatory cells, also play important roles in cutaneous tissue repair. Here, we describe the characteristics of wound healing, concentrating on the production and functions of EVs derived from specific skin cells, and provide new ideas for wound therapy using EVs.

## Highlights

- This article reviews EVs secreted by cells residing in skin tissue and their roles in wound healing.
- EVs derived from skin tissue cells reflect cell–cell cross-talk status in the microenvironment during the healing process.
- EVs provide new insights into the treatment of cutaneous injury and the current challenges, as well as pending questions in EVs application are also involved.

## Background

The skin is a multilayered primary defense organ, comprising various cell types that intercommunicate to make it an effective barrier. Intimate intercellular communication in the skin is necessary for effective surveillance. The skin is the most vulnerable when exposed to the outside environment. Cutaneous wounds can be caused by trauma, burns and chronic diseases, and pose a great therapeutic challenge worldwide [1,2]. After cutaneous injury, the cells and microenvironment at the wound site respond rapidly to promote healing. Dynamic cellular events following cutaneous injury rely on bidirectional cell–cell communication for efficient wound healing. As intercellular mediators, skin cells transmit signals via immediate contact or release of soluble factors, including proteins, genetic material, such as microRNAs (miRNAs), and extracellular vesicles (EVs), transporting bioactive molecules to adjacent and distant cells for communication and organ regulation [3]. To some extent, EV-borne molecular signals drive the cross-talk between different cellular compartments in a way that directly determines the wound healing outcome. EVs originating from specific cells are being explored for their involvement in intercellular skin communication. In this review, we focus on the role of EVs secreted from different cells residing in skin tissues during the wound healing process. Furthermore, we summarize the recent research progress on EVs and emphasize their clinical relevance to facilitate the development of novel therapeutic strategies.

## Review

### Wound healing

Wound healing is a highly coordinated process in which numerous cell types are extensively mobilized to restore the injured tissue. It occurs via distinct overlapping phases: hemostasis, inflammation, proliferation and remodeling. In the early stages of the wound, there are varying degrees of tissue necrosis, blood vessel rupture and bleeding in the wound area. Within a few hours, an inflammatory response occurs, which is followed by local redness and swelling. Neutrophils predominate during early leukocyte infiltration, releasing toxic granules, producing an oxidative burst, initiating phagocytosis, and generating neutrophil extracellular traps (NETs) to eliminate infections [4]. Macrophages predominate approximately three days later. Fibrinogen promptly solidifies to form clots that stop the bleeding and protect the wound. Subsequently, the skin and subcutaneous tissue around the edges move toward the center to shrink the wound. Granulation tissue then grows from the bottom and edges of the wound to fill it, collagen fibers proliferate to form a scar. Eventually, the epidermis is re-epithelized [5].

During the entire repair process, various cell groups cooperate, contributing to the coordination of individual events and facilitating temporary and spatial control [6]. Monocytes, platelets, inflammatory cells, epidermal cells and dermal cells interact with each other to promote wound healing. Macrophages are conducive to cell growth, differentiation, extracellular matrix (ECM) formation, and remodeling by serving as reservoirs for the release of growth factors [7]. During transition from the inflammation phase to the granulation tissue phase, cellular interactions gradually become dominated by the interplay between keratinocytes and fibroblasts. Epidermal keratinocytes can interact with dermal fibroblasts either via soluble mediators or secreted vesicles that carry signaling molecules to transfer information between the two cell types [8]. Hence, the whole repair process is not a single-player process but requires cell–cell cross-talk and interaction between distinct cell types, in which EVs are most likely to be responsible for pathophysiologic message exchange.

Notably, the epithelial–mesenchymal transition (EMT) process during which epithelial cells acquire mesenchymal fibroblast-like characteristics, is an indispensable event involved in both wound healing and scar formation. Generally, EMT includes three diverse subtypes: type-1, type-2 and type-3 EMT. Type-1 EMT is associated with embryogenesis and organ development. Type-3 EMT occurs in neoplastic cells and promotes the growth of localized tumors via genetic and epigenetic modifications. The EMT linked to wound healing, tissue regeneration and organ fibrosis is classified as type-2 EMT [9]. In the early proliferative phase of wound healing and tissue repair, fibroblasts migrate into the wound and secrete collagen-rich ECM to stimulate granulation tissue formation. In the late period of the proliferative phase, fibroblasts are activated and differentiated into contractile myofibroblasts to promote wound contraction. Type-2 EMT, to some extent, can be understood that non-fibroblasts have the capability of trans-differentiation into fibroblasts and myofibroblasts with the profibrotic and proinflammatory activity to participate in wound healing and tissue repair. Type-2 EMT, to some extent, can be interpreted as that non-fibroblasts have the capability of trans-differentiation into fibroblasts and myofibroblasts with the profibrotic and proinflammatory activity to participate in wound healing and tissue repair. However, myofibroblasts act as double-edged swords, excessive ECM secretion can cause adverse scarring [10]. After the acute and moderate injury, the healing event is considered as remedial action. In the continuing chronic injury and inflammation, anomalous generation of myofibroblasts stimulates a progressive fibrosis, leading to the structure and function destruction of organ and tissue [11]. It is suggested that anti-EMT strategies could be used for antifibrotic/scar therapy during the specific period of repair.

### Extracellular vesicles

EVs are lipid bilayer-encapsulated nanoparticles with a size of 50–1000 nm [7] that can be released by all cell types and found in various body fluids [12], including blood, urine, saliva, breast milk, amniotic fluid, ascites, cerebrospinal fluid, bile and semen [13]. Increasing evidence indicates that they play an essential role not only in the regulation of normal physiological processes, such as stem cell maintenance, tissue repair, immune surveillance and blood coagulation, but also in the pathology of several diseases [14–17].

#### Subpopulations and biogenesis

EVs cover various subtypes of cell-released membranous structures. The classification standards for EVs subtypes have not yet been unified. According to MISEV2018 [18], EVs can be divided into medium/large EVs (>200 nm) and small EVs (sEVs < 200 nm) based on their physical properties. EVs can also be classified based on their origin (e.g. apoptotic bodies with 50–1000 nm diameter, microvesicles with 100–1000 nm diameter, exosomes with 40–160 nm diameter, and average 100 nm diameter) or biological composition (such as surface protein CD63^+^ EV). Additionally, prevailing conditions are used to distinguish EVs as large oncosomes, hypoxic EVs and podocyte EVs. There are many EVs whose functions and composition have not yet been characterized [19,20].

Exosomes are widely studied, classical exosomes formed by the endosomal system that are formed via three stages: the plasma membrane of the cell invaginates for the first time to form endocytic vesicles, multiple endosomes fuse to form early endosomes, and intraluminal multivesicular bodies (MVBs) are formed in the early endosomes that enclose the intracellular materials. Lastly, MVBs fuse with the plasma membrane, releasing intraluminal vesicles (ILVs), namely exosomes, into the extracellular space [21]. Endosomal sorting complex required for transport (ESCRT) drives the formation of MVBs and ILVs. ESCRT comprises approximately 30 proteins that assemble into four complexes (ESCRT-0, -I, -II and -III), which are linked to cargo recruitment and inward invagination of the late endosomal membrane [22]. ESCRT-0 complex recognizes and sequesters ubiquitinated transmembrane proteins in the endosomal membrane, whereas the ESCRT-I and II complexes are responsible for membrane deformation into buds with sorted cargo. ESCRT-III can be activated by ESCRT-II and subsequently drives vesicle scission [23]. In addition, ESCRT-independent pathways also regulate the inward budding of the MVB. Sphingomyelinase hydrolysis and ceramide formation are major processes, which can stimulate the spontaneous negative curvature of MVB membrane to form ILVs. Then the MVB fuses with the cellular membrane and releases the ILVs, named exosomes or ‘exosome-like vesicles’ [24,25].

**Figure 1.**
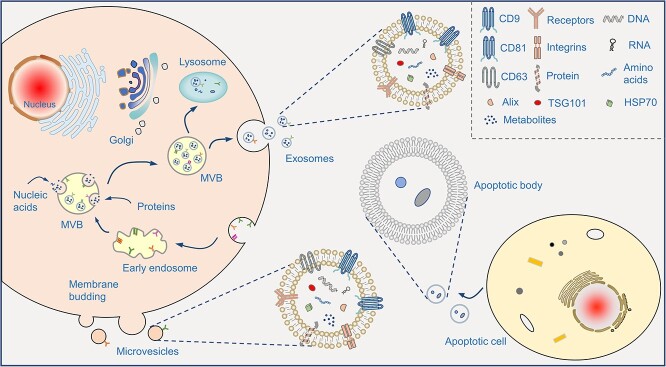
The biogenesis, components of EVs. Exosomes are derived from the fusion of multivesicular bodies (MVB) and plasma membranes. Microvesicles or microparticles are formed after direct outward budding of plasma membranes. EVs contain membrane proteins and cytosolic components (nucleic acids and proteins) and release the cargos to extracellular matrix. *EV* extracellular vesicle

The formation process of other EV types is as follows: apoptotic bodies are usually released in the process of cell apoptosis, formed by the wrinkling and invagination of the cell membrane and the subsequent division and wrapping of the cytoplasm [26]. Microvesicles (MVs) are related to the asymmetric distribution of phosphatidylcholine and sphingomyelin in the outer membrane, and phosphatidylserine and phosphatidylethanolamine in the inner membrane. This asymmetric distribution is mainly maintained by three types of proteins: flippase, floppases, and phospholipid scramblase. However, cytoplasmic calcium influx blocks phospholipid asymmetry by activating phospholipid scramblase. This activation leads to the redistribution of phospholipids in the bilayer of the cell membrane, encouraging the budding of the cell membrane [27] (Figure 1).

### Components

EVs are enriched in various bioactive substances, mainly proteins, nucleotides (DNA, RNA, mRNA, miRNA, long non-coding RNA [lncRNA] and circular RNA), and lipids [28]. In recent years, with the rapid development of new detection technologies, bioactive components of EVs have been identified. Proteins are notable components of the EV cargo. EV proteins can be divided into two types: ubiquitous proteins involved in the formation of their structures, such as cytoskeleton components and members of the quaternary transmembrane protein superfamily, and those associated with their cell origin [29]. EVs contain several types of nucleic acids, including guideDNA [30], mitochondrial DNA [31] and RNA [23]. Moreover, EVs are abundant in cholesterol, phospholipids, phosphatidylethanolamine and other lipids, which are involved in the biosynthesis and uptake of EVs [32]. Various sorting mechanisms of EV cargo have been described, including the ESCRT machinery [33], syntenin-Alix pathway [34], tetraspanins [35], cytoskeleton [36], lipids [37], and arrestin domain-containing protein 1 [38]. These sorted pieces of machinery enrich specific sets of cargoes into EVs, and their consumption can eliminate the production of a defined subpopulation of EVs [39].

### EVs in regenerative medicine

Regenerative medicine is aimed to functionally regain or restore tissue and organs from damage, or to form them when they are lost [40]. Cell-based therapies are developed as effective means, in which viable cells, such as stem cells, progenitor cells, inflammatory cells, cartilage cells, islet cells and liver cells, are used for tissue repair [41–43]. Among these, stem cells are the most widely mentioned as they can differentiate into various cell types to mediate tissue repair and regenerative process [44]. In recent years, EVs are emerging as cell-free alternatives to cell therapies, which are exceptionally attractive due to their intrinsic biocompatibility, biodegradability, low toxicity, low immunogenicity and engineered modification [45]. EVs have been explored for *in vitro* and *in vivo* studies in various diseases treatment, such as the cardiovascular regeneration, nerve, kidney, liver, lung and skin tissue regenerative therapy [46]. Many clinical trials have also been conducted (https://clinicaltrials.gov/). The key molecules in EV-mediated tissue repair and regeneration processes include miRNAs, mRNAs and proteins. Several signaling pathways involved in regenerative processes, such as mitogen-activated protein kinase, Wnt/b-catenin, PI3K/Akt, Notch, TGF-β/Smad, STAT and Hedgehog signaling, CaMKII, and Efna3 signaling, have been identified [47–50]. Given their advantages of nano-size and biocompatibility, EVs can cross natural biological barriers, such as the blood–brain barrier, which is a potential strategy for brain and central nervous system injury. In addition, EVs-integrated biomaterials can encourage bone regeneration [51]. EVs can also be loaded with biological macromolecules, short peptides, miRNAs, siRNAs and small molecule drugs to promote tissue repair capabilities [52]. For example, the anti-inflammatory drug, curcumin can be encapsulated into EVs to treat various tissue injuries, such as brain, lung and cutaneous injuries, and cerebral ischemia [53–56]. Catalase-loaded exosomes protect the neurons in a mouse model [57]. Some growth factors, such as TGF-β, fibroblast growth factor, vascular endothelial growth factor, and glial cell-derived neurotrophic factor are abundant in mesenchymal stem cell-derived EVs (MSC-EVs), and their delivery contributes to multiple tissue injuries [58,43]. Although this potential has not yet been well characterized, with the growing knowledge of functional roles of EVs, their applications in regenerative medicine will continue to expand.

### EVs in cutaneous injury

**Figure 2.**
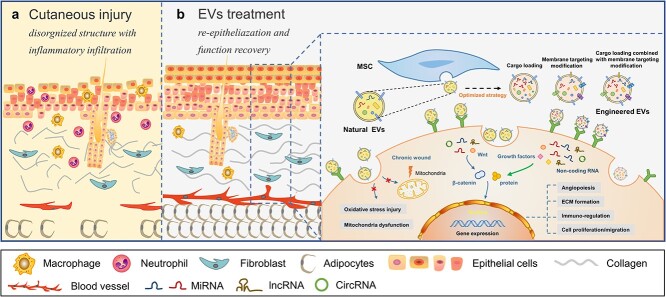
The diagram of EVs repair cutaneous injury and mechanism. **(a)** Cutaneous injury. The entire structure of the skin is destroyed, accompanied by an inflammatory response. In chronic wounds such as diabetic ulcers, the skin tissue is severely damaged, the epidermis is incomplete, dermal fibers are broken, blood vessel formation disorders with continuous inflammatory infiltration. **(b)** EVs treatment. EVs promote epidermal and dermal architecture and function recovery and angiogenesis and play an immunomodulatory role through RNA, such as microRNA and circRNA, as well as proteins and growth factors. Engineered EVs repair cutaneous injury better. *EV* extracellular vesicle, *ECM* extracellular matrix, *MSC* mesenchymal stem cell

Cutaneous injury is a common clinical issue, including acute or chronic cutaneous injury due to trauma, burns and diabetes. For chronic severe injury with continuous inflammation, there is a lack of better therapies. Increasing evidence has revealed that EVs are promising therapeutics and drug delivery platforms [59]. It is widely accepted that MSC-EVs are a promising candidate that aids and accelerates wound healing. Due to their extensive effects, MSC-EVs affect a variety of cells associated with wound healing, including keratinocytes, dermal fibroblasts, inflammatory cells and vascular endothelial cells. MSC-EVs participate in the entire healing process by regulating the immune response and inflammation, accelerating proliferation and re-epithelialization of skin cells, modulating ECM remodeling, and promoting angiogenesis [60,61] (Figure 2). EVs derived from other cells, such as plasma endothelial cells [62], induced pluripotent stem cells (iPSCs) [63], perivascular cells [64] and platelet [65]. In addition, in the context of the skin, studies have discovered in *ex vivo* sections of the human papillary dermis [66], sites of age-related cutaneous disorders [67], injury [68], and stroma of human skin carcinomas [69]. Moreover, *in vitro* vesicular cross-talk has been observed between several types of skin cells, including keratinocytes, melanocytes, human dermal fibroblasts (HDFs), dermal papilla cells (DPCs), outer root sheath (ORS) cells of the hair follicle, and microvascular endothelial cells [70–72]. How are EVs from specific skin tissue cells that participate in cutaneous repair linked to wound healing? To date, only a few studies have investigated the therapeutic potential of a population of specific skin cells that reside in or participate in wound healing (Table 1).

**Table 1:** Roles of extracellular vesicles derived from special skin tissue cells in wound healing

**EVs sources**	**Interacting cells**	**Cargoes**	**Mechanism(s) and effect(s)**	**Reference**
Epidermal keratinocyte	Melanocyte	miRNA-203	Raised the production of Tyr and Rab27a proteins and regulates pigmentation	[56]
		miRNA-3196	Augmented the production of MITF-M and Rab27a and activates the MITF pathway to upregulate melanin production	[56]
		miR-330-5p	Downregulated Tyr to suppress melanocyte pigmentation	[57]
	Fibroblasts	miRNA-21	Induced fibroblast function through MAPK/ERK signaling, amplified immune response, and reduced PTEN and RECK expression	[60]
	Vascular endothelial cells	/	Regulated the expression of wound healing-related genes in DFs and induced fibroblast-mediated endothelial tube formation	[62]
	keratinocyte	/	Enhanced DFs and KCs migration via the MAPKinase pathway	[63]
	Neutrophil	Special proteins	Promoted proinflammatory factor production via NF-κB and p38 MAPK signaling pathways	[64]
	Macrophage	Specific surface N-glycan, miRNA	Facilitated the uptake of EVs by macrophage at the wound-edge	[65]
	T cells	miR-381-3p	Activated T-bet and RORγt transcription, inducing Th1 and Th17 polarization	[67]
	HFSCs	/	Triggered β-catenin and expression of AXIN2, induced HFSCs proliferate and hair growth	[68]
Epidermal stem cell	Epidermal cells	Proteins	Promote epidermal cell growth via various proteins involving epidermis development	[72]
	Myofibroblast	miR-425-5p miR-142-3p	Inhibited myofibroblast differentiation via reducing the TGF-β1 expression	[73]
	Fibroblasts, macrophages	miRNAs	Augmented wound cell proliferation and M2 Mφ polarization through PI3K/AKT and TGFβ signaling pathways possibly	[74]
Dermal fibroblast	HUVECs	/	Accelerated diabetic cutaneous wound healing through the Akt/β-catenin pathway	[79]
	DFs	/	Increased DFs proliferation, inhibited production of ROS induced by UVB radiation due to upregulation of GPX-1 expression possibly	[80]
	HDFs	miRNA cargos	Restored the function of aged HDFs and ameliorated skin photoaging	[82]
	Keratinocyte	/	Regulated epidermal homeostasis by reflecting cellular status	[84]
		miR-23a-3p	Expedited scratch closure of KCs and regulated skin homeostasis	[8]
	DPs, ORCs	Wnt3a	Promoted cells function and elongation of the hair shaft via Wnt/β-catenin pathway	[85]
	DPs	Frizzled4	Stimulated HF growth through DP-secreted Norrin	[86]
	Endothelial cells	Proteins	Increased endothelial cells growth, migration and capillary formation	[89]
	DFs	PLGF-1	Stimulated collagen production and wound healing through PLGF-1 signaling potentially	[90]
Dermal papilla cell	Hair matrix cells	/	Facilitated proliferation of hair matrix cells and hair growth via upregulating Wnt3a and β-catenin downregulating of inhibitory molecule BMP2	[93]
	DPCs	/	Increased the expression of growth factors (IGF-1, KGF and HGF) and promotes hair growth	[94]
	Outer root sheath cells	/	Enhanced ORSCs proliferate and migrate to regulate HF development, and stimulated the expression of β-catenin and Shh	[95]
	MxCs, ORSCs	miR-140-5p	Accelerated HF elongation and cell proliferation through BMP/TGF-β signaling pathways	[96]
	HFSCs	miR-22-5p	Regulate HFSCs proliferation and differentiation via miR-22-5p-LEF1 axis	[97]
Macrophage	Endothelial cells	/	Attenuated secretion of proinflammatory cytokine, and promoted proliferation and migration of endothelial cells to improve angiogenesis and re-epithelialization	[106]
		miR-130a, VEGF, Wnt3a	Increased endothelial cellular proliferation, migration and new vasculature formation	[107]
	Epithelial cells	miR-590-3p	Reduced inflammatory signals and promoted epithelial regeneration via LATS1/YAP/β-catenin signaling axis	[108]
	DFs	lncRNA-ASLNCS5088	Activated fibroblast and may cause hypertrophic scar formation via adsorbing microRNA-200c-3p, causing increased GLS and α-SMA expression	[109]
		LINC01605	Promoted proliferation, migration and invasion of DFs via miR-493-3p/AKT signaling pathway	[110]
	DPCs	/	Induced DPCs proliferation and hair growth	[71]
Endothelial progenitor cell	Vascular endothelial cells	/	Accelerated re-endothelialization by enhancing endothelial function	[117] [118]
		/	Advanced wound healing by promoting angiogenesis through Erk1/2 signaling	[119]
		miRNA-221-3p	Increased expression of angiogenesis-related factors via cell cycle, AGE-RAGE and the p53 signaling pathway probably	[120]

*Tyr* tyrosine, *MITF-M* microphthalmia-associated transcription factor melanocyte isoform, *HFSCs* hair follicle stem cells, *DPCs* dermal papilla cells, *ORSCs* outer root sheath cells, *HF* hair follicle, *MxCs* hair matrix cells, *HUVECs* human umbilical vein endothelial cells, *HDFs* human dermal fibroblasts, *EV* extracellular vesicle, *α-SMA* alpha-smooth muscle actin, *DF* dermal fibroblasts

### EVs derived from epidermal keratinocytes

Keratinocytes are highly specialized epithelial cells that play a major role in epidermal restoration after injury through proliferation and re-epithelialization [73]. Within cutaneous tissues, keratinocytes interact with small populations of other cell types, including melanocytes, Langerhans cells, intraepithelial lymphocytes and fibroblasts. Chavez-Muñoz et al. primarily reported that keratinocytes can produce exosomes through which some intracellular proteins such as stratifin, with matrix metalloproteinase (MMP)-1 stimulating activity for fibroblasts, are externalized into the keratinocyte microenvironment [74]. Keratinocyte-derived EVs (KC-EVs) contain a variety of bioactive molecules, such as nucleic acids, metabolic enzymes, cytoskeletal proteins, signaling proteins, trafficking proteins and adhesion proteins, and have significant physiological consequences on target cells [75,76].

Melanocyte–keratinocyte interactions are a well-studied system in which melanocyte activity, such as pigment production and pigment transfer, is assisted by EVs [77]. The study demonstrated that miRNA-203 and miRNA-3196 are two major functional molecules. KC-EVs conveyed miRNA-203 increases the production of tyrosine (Tyr) and Rab27a proteins, which subsequently induce Tyr activation and pigmentation. KC-EVs shuttled miRNA-3196 augments the production of microphthalmia-associated transcription factor (MITF) melanocyte isoform (MITF-M) and Rab27a, which then activates the MITF pathway to upregulate melanin production [78]. Liu et al. demonstrated that keratinocytes secrete exosomes carrying miR-330-5p and decrease pigmentation in melanocytes [79]. It has also been reported that keratinocyte-derived MVs can mediate ultraviolet B radiation-induced systemic immunosuppression [80]. The interaction between keratinocytes and melanocytes reveals a potential mechanism of pigment production.

Additionally, the ECM of the epidermis is enriched with KC-EVs, and interactions with integrins expressed by intact cells regulate multiple cellular activities, such as adhesion, migration, differentiation, apoptosis and expression of specific genes [81,82]. It is hypothesized that EVs secreted by keratinocytes and incorporated into the ECM may deliver their cargo, including integrins, to dermal fibroblasts during the healing process. Li et al. found that human keratinocyte-derived MVs delivered miRNA-21 and promoted skin wound healing in diabetic rats by facilitating fibroblast function and angiogenesis [83]. EVs can also affect the gene expression in fibroblasts. For example, EVs derived from epidermal cells significantly regulate genes encoding MMP-1 and -3, IL-6 and IL-8, and genes associated with TGF-β signaling in fibroblasts. In addition, the presence of MV-like vesicles during active keratinocyte migration and early stages of granulation tissue organization in human wounded skin [8,84]. In addition, it has been proved that MV-like vesicles are present in the early stages of active keratinocyte migration and granulation tissue organization in injured human skin. Another study identified that HaCaT cell-derived EVs expedite the migration and proliferation of human keratinocytes and fibroblasts and may advance wound healing via activation of the mitogen-activated protein kinase pathway [85].

Moreover, EVs are emerging as mediators of intercellular communication between keratinocytes and immune cells. Cytokine-treated keratinocytes secrete exosomes that promote neutrophil pro-inflammatory factor production, containing IL-6, IL-8 and TNF-α [86]. Wound macrophages selectively absorb exosomes derived from wound-edge keratinocytes because of the specific surface N-glycan and miRNA packaging, inducing a wound inflammation response. This indicates close cross-talk between keratinocytes and macrophages through exosome-delivered miRNAs [87,88]. Jiang et al. demonstrated that EVs generated by cytokine-treated KCs promoted the polarization of Th1 and Th17 cells in psoriasis [89]. This highlights the importance of EVs as a communication strategy between KCs and T cells under psoriasis-like conditions. Cutting off these lines of communication by either inhibiting EVs secretion or blocking functional cargo could be a useful therapeutic strategy in patients with psoriasis and other inflammatory diseases. KC-EVs also participate in hair growth, and a study found that EVs derived from fisetin-treated KCs mediate hair growth promotion [90]. It has been suggested that exosomes can be considered as natural mediators that could be involved in hair cycle regulation and serve as promising delivery vehicles for improving skin and hair regeneration because of their potential to target various molecular processes and cells.

### EVs derived from epidermal stem cells

Keratinocytes proliferate from the basal cells of the innermost layer of the skin (stratum basal) [91]. Epidermal stem cells (ESCs) are attached by hemidesmosomes to the stratum basal, maintain skin homeostasis and hair regeneration, and provide the original power for re-epithelialization after injuries [92]. ESCs are morphologically similar to KCs, but have specific identification markers. These are of particular interest because they are numerous and accessible. Additionally, ESCs are easy to obtain without potential ethical and political issues compared with embryonic stem cells [93]. ESCs also possess a strong paracrine function; therefore, their effects on epidermal cells, dermal cells and angiogenesis in wound healing should not be ignored.

Leng et al. demonstrated that ESC-EVs were beneficial in epidermal cell growth and classical stemness regulators, and that Wnt signaling was always involved [94]. Duan et al. determined that ESC-derived exosomes enhance the wound healing rate and reduce scar formation in rats by delivering miR-425-5p and miR-142-3p [95]. Recent research has shown that exosomes originating from ESCs could improve diabetic wound healing and both ESCs and ESC-Exos have an equal effect. This indicates that ESC-Exo treatment may be a promising and technically advantageous stem cell-based therapeutic strategy [96].

### EVs derived from dermal fibroblasts

Cutaneous injury repair depends on the interaction between cells and the ECM. Fibroblasts are the main force in damage repair and ECM formation. Under the stimulation of several chemokines, fibroblasts migrate from the wound perimeter to the wound surface and secrete a large number of ECM components, such as collagen, fibronectin, laminin, vitronectin and proteoglycan. The capacity of dermal fibroblasts (DFs) in wound healing has been well clarified; however, their function seems to be underestimated. Fibroblasts are mesenchymal cells [97], which are gradually considered to be convenient alternatives to MSCs because of their common characteristics, such as expression of the same mesenchymal markers and multi-differentiative potential toward mesodermal lineage, and anti-inflammatory, immunomodulatory and regenerative effects [98,99]. Another explanation is that cultured DFs are mixed-cell populations, including stem cells at different developmental stages [100]. Their therapeutic potential is similar, it should be noted that DFs are more accessible than MSC and are easier to expand *in vitro*. Thus, the utilization of DFs may be more practical in clinical applications.

As active mediators of both the inflammatory and proliferative phases of wound healing, DFs communicate with all cell types in the wound bed through direct contact or in autocrine and paracrine manners [101]. EVs are key components of paracrine signaling and are responsible for communication between cells during wound healing. DFs-EVs have not been well investigated as they are generally accepted as terminally differentiated cells. Our previous study found that exosomes derived from autologous DFs could reverse the damage caused by high glucose levels in human umbilical vein endothelial cells (HUVECs) *in vitro* and promote diabetic cutaneous wound healing through the Akt/β-catenin pathway [102]. Deng et al. demonstrated that both MSC-EVs and DFs-EVs showed antioxidant activity that could reduce the intracellular levels of reactive oxygen species (ROS) induced by UVB irradiation, although DFs-EVs were initially used as a negative control [96]. Interestingly, Wang et al. indicated that exosomes derived from ESCs (ESC-Exo) accelerated wound healing when compared to fibroblast exosomes (FB-Exo) and phosphate buffered saline (PBS) controls [103]. From the experimental results, FB-Exo also has an effect but is not as effective as ESC-Exo. Due to the conditions and standardization of research, it is difficult to conclude which origin of exosomes works the best. Furthermore, EVs derived from three-dimensional cultured DFs are also able to induce efficient collagen biosynthesis and ameliorate inflammation in UVB-induced skin injury, even better than those isolated from two-dimensional cultured DFs [104]. Changes in growth conditions and the culture environment can alter the state of cells, including their paracrine function. By changing the growth environment or adding stimulation factors, cells can transform to a better state and secrete EVs with higher yields and functions to achieve a better therapeutic effect, which is also a research hotspot.

EVs facilitate intercellular communication and enable cells to exchange proteins, lipids and nucleic acids [105]. The dermis and epidermis are in close contact with each other; therefore, what is the role of EVs as a means of communication? Choi et al. investigated whether human dermal fibroblast-derived EVs (HDFs-EVs) affect the homeostatic regulation of keratinocytes and found that senescent fibroblasts produced more EVs than young HDFs; however, these EVs attenuate the dermal effect on keratinocyte differentiation and evoke the pro-inflammatory cytokine IL-6 [106]. Similarly, senescent fibroblasts-derived EVs accelerated the scratch/gap closure of keratinocytes by delivering miR-23a-3p [107]. In addition, HDF-EVs can increase the ability of human DP and ORS cells, thereby promoting hair growth in cultured human hair follicles [108]. EVs from activated DFs stimulate hair follicle growth via DP-secreted norrin [109]. During wound healing, myofibroblasts shrink the wound by contracting the wound edges and secreting a large quantity of extracellular matrix to connect with the surroundings [110]. Myofibroblasts, particularly those existing in the wound, also produce MVs as mediators of intercellular communication to facilitate ECM formation and neovascularization [71]. The release of MVs by wound myofibroblasts brings new perspectives to the field of communication between cells during the normal healing process [111].

### EVs derived from dermal papilla cells

Hair is a defining feature of mammals and has critical functions, including protection, production of sebum, apocrine sweat and pheromones, social and sexual interactions, thermoregulation, and the provision of stem cells for skin homeostasis, regeneration and repair [112]. Hair follicles and hair growth are critical indicators of skin recovery after injury. Hair DPCs are specialized mesenchymal cells that reside in the DP located at the bottom of the hair follicles [113]. DPCs serve as signaling centers in hair follicles and regulate hair formation and cycling via paracrine secretion. DPC-derived EVs (DPC-EVs) are critical for hair regeneration.

Numerous studies have demonstrated that DPC-EVs contribute to hair growth. For instance, research has revealed that the sustained release of DPC-EVs from injectable microgel promotes hair growth, which may be due to the upregulation of hair growth-promoting signaling molecules, such as Wnt3a and β-catenin, and downregulation of the inhibitory molecule BMP2 [114]. Another finding indicated that DPC-EVs encourage hair growth and regeneration by modulating the activity of follicular dermal and epidermal cells and augmenting the hair-inductive capacity of cultured DP spheres [115]. *In vitro*, DPC-EV treatment enhances the ORS proliferation and migration and stimulates the expression of β-catenin and sonic hedgehog [116]. Correspondingly, Chen et al. also demonstrated that EVs containing miR-140-5p from human papilla cells stimulate hair growth by promoting the proliferation of ORS and hair matrix cells [117]. DP also regulate stem cells; for example, Yan et al. found that miR-22-5p derived from DP-EVs mediates hair follicle stem cell proliferation and differentiation [118]. Additionally, DPC-EVs also increase hair-inductive gene expression in adipose stem cells via β-catenin activation [119]. Overall, DPC-EVs represent a therapeutic target for damage repair, especially for hair growth after injury.

**Figure 3.**
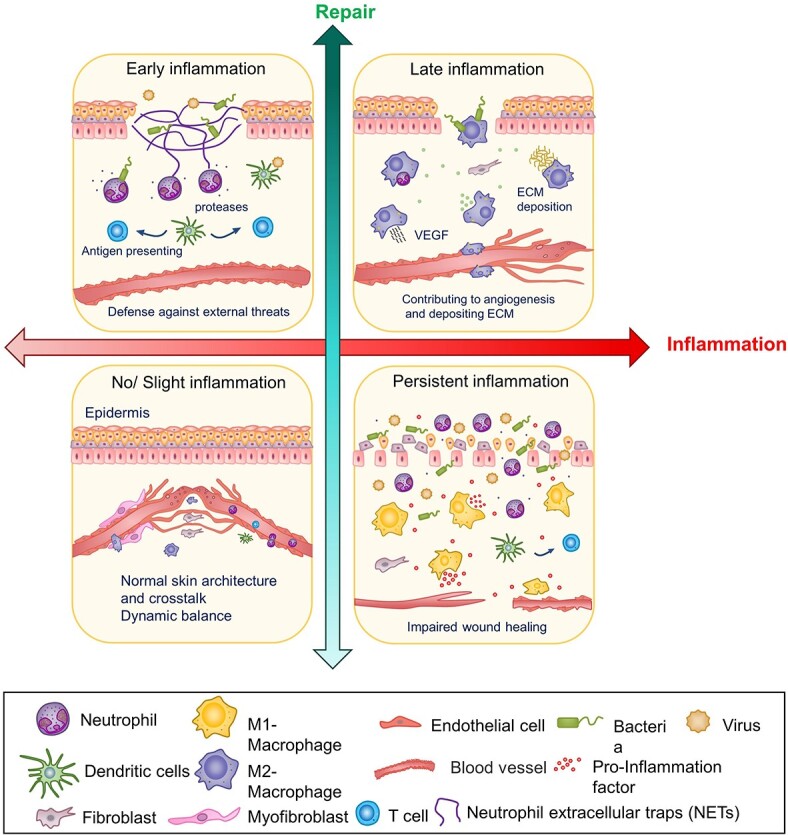
Quadrants reflecting inflammation and cutaneous injury repair. Moderate inflammation contributes to repair, but excessive inflammation has the opposite effect. The first quadrant represents the early stages of inflammation. Neutrophils play an antibacterial and threatening role, dendritic cells (DCs) are antigen presenting cells that are involved in priming T-cell responses. The second quadrant represents the late stage of inflammation, in which M2 macrophages play a dominant role. The third quadrant represents the normal homeostasis of the skin when there is no injury or slight inflammation. The fourth quadrant represents excessive inflammation in pathological conditions, where the action of a range of inflammatory cells and proinflammatory factors makes wound healing difficult. *ECM* extracellular matrix, *VEGF* vascular endothelial growth factor

**Figure 4.**
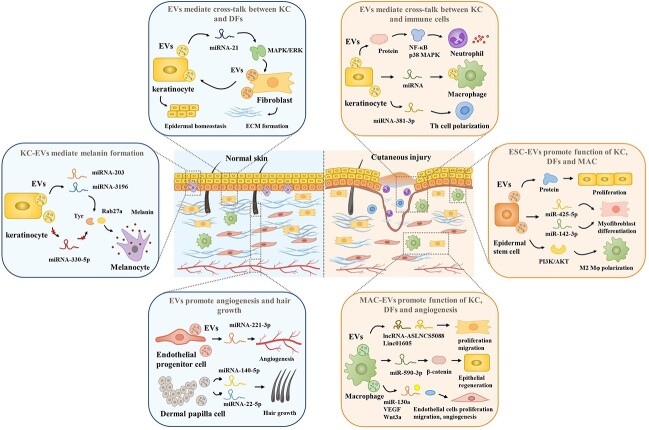
Diagrammatic illustration of EVs derived from different types of cells, including keratinocyte, epidermal stem cells, fibroblast, macrophages, dermal papilla cells and endothelial progenitor cells. These cells are related to cutaneous injury and their cross-talk plays significant roles in wound healing. *ECM* extracellular matrix, *VEGF* vascular endothelial growth factor, *EV* extracellular vesicle, *KC* keratinocyte, *MAC* macrophage, *DF* dermal fibroblasts

### EVs derived from macrophages

In wound healing, inflammation is a major physiological response and a necessary period of transition, which is orchestrated by cells (such as leukocytes, stem cells and tissue-resident cells) and the molecular messengers they secrete, including cytokines, growth factors and exosomes [120]. Immune cells play a prominent regulatory role in the inflammatory and proliferative phases of skin healing. A moderate inflammatory response promotes damage repair; however, excessive inflammation may cause further damage. Among the immune cells in the wound, macrophages are key players that facilitate the inflammatory proliferation phase transition. Nevertheless, macrophages show the opposite effect due to their subtypes: classically activated M1 with pro-inflammatory properties and alternatively activated M2 exhibiting anti-inflammatory and wound healing functions [121]. In the early stages of wound healing, macrophages are usually polarized to M1 macrophages, which are microbicidal and pro-inflammatory [122]. With the resolution of inflammation, macrophages transform into M2 macrophages, which secrete vascular endothelial growth factor (VEGF) to support angiogenesis [123,124]. During the proliferation stage, macrophages also interact with DFs and induce them to myofibroblast transformation, increasing ECM deposition [125,126]. An overreactive inflammatory response during the inflammatory phase or excessive deposition of ECM during the proliferation phase causes abnormal scar formation [127]. Therefore, the study of inflammatory cells, particularly macrophages, is indispensable (Figure 3). Macrophage-derived exosomes have been shown to accelerate wound healing through anti-inflammatory effects and by inducing endothelial cell proliferation and migration to improve angiogenesis and re-epithelialization in diabetic wounds [128]. Another study also indicated that macrophage-derived EVs (MAC-EVs) promote angiogenesis *in vitro* and accelerate new vasculature formation *in vivo* and found that VEGF, Wnt3a and miR-130a were abundant in MAC-EVs, even more than in parental cells [129]. M2 macrophage-derived exosomal miR-590-3p reduces inflammatory signals and promotes epithelial regeneration [130]. Macrophages also stimulate DFs activity, and activated macrophages (M2) manipulate fibroblasts to differentiate into myofibroblasts with active biological functions and proliferation, which may cause a hypertrophic scar. Chen et al. demonstrated that lncRNA-ASLNCS5088 derived from M2 macrophage exosomes orchestrates fibroblast activation, and its blockade by DW4869 dampens this effect [131]. Similarly, another study indicated that the inhibition of linc01605-enriched exosome generation in M2 macrophages impairs M2 macrophage-induced proliferation, migration and invasion of HDFs [132]. This finding further supports the dual role of macrophages. Furthermore, engineered EV-mimetics from macrophages facilitate hair growth in mice and encourage human hair follicle growth [133]. The complexity of macrophages in wound healing makes them more difficult to study, and the research is not sufficiently in-depth, which also provides a possibility for future research.

### EVs derived from endothelial progenitor cells

Endothelial progenitor cells (EPCs), a particular type of stem cells, represent a heterogeneous population of resident mononuclear cells that originate from the bone marrow (BM) [134]. Although EPCs do not originate in the skin, they influence skin wound healing as precursors of vascular endothelial cells [135]. Vascular formation is an essential part of skin wound healing, which provides oxygen for the cells and microenvironment at the wound site, and is conducive to the formation of granulation tissue. EPCs can secrete paracrine factors such as growth factors, cytokines, chemokines and bioactive lipids that influence cell biology in damaged tissue, among which EVs are crucial bioactive factors [136,137]. EPC-EVs have potential therapeutic applications in tissue repair and regeneration, such as diabetic foot ulcers, kidney disease and bone healing [138,139]. Studies have shown that exosomes derived from EPCs attenuate vascular repair and accelerate re-endothelialization by enhancing endothelial function [140]. EPC-derived exosomes have been reported to facilitate skin wound healing by positively modulating endothelial cell function [141]. After local injection of EPC-EVs into the skin wounds of diabetic rats, Zhang et al. [142] discovered that EPC-EVs could significantly improve wound healing and accelerate angiogenesis in damaged areas by modulating extracellular signal-regulated kinase 1/2 signaling. Xu et al. [143] also identified exosomes isolated from murine BM EPCs that accelerate skin wound healing in both control and diabetic mice by transferring miRNA-221-3p. Hassanpour et al. found out that the diabetic state could affect the CD63-Alix-Rab27a signaling pathway, thereby reducing the formation, transportation and fusion of EVs in EPCs [144]. Those results underline the role of EPC-exosomes and suggest new potential approaches to the therapy for diabetic skin wounds (Figure 4).

### Open questions and future directions

Accordingly, EVs derived from both MSCs and specific skin tissue cells are involved in wound healing and can be used as promising agents for damage repair. However, several challenges and questions remain to be addressed.

#### Skin cell-EVs *vs* MSC-EVs

MSCs have diverse sources, includingBM, adipose tissue, umbilical cord, umbilical cord blood, dental pulp, synovial fluid, amniotic fluid, placenta, Wharton’s jelly and body fluids [145]. In addition, MSCs have immunomodulatory properties to regulate various cells involved in immune responses [146]. Clinical trials have indicated that MSC-based therapy could effectively improve burn wound healing, and treat hypertrophic scars [147,148]. Urine-derived stem cells have been successfully used for skin, bone and articular cartilage repair [149]. EVs are important effectors of MSCs in paracrine activity, emerging as the most promising candidates for tissue repair and regenerative medicine. Our previous research found that hucMSC-exosomes containing Wnt4 promoted cutaneous wound healing in a rat skin burn model [150]. Exosomes originating from both human umbilical cord blood mesenchymal stem cells and human BM-MSC effectively stimulate cutaneous wound healing by inhibiting the TGF-β signaling pathway [151,152]. Other studies indicated that adipose mesenchymal stem cell-derived exosomes accelerate skin wound healing via the lncRNA H19/miR-19b/SRY-box transcription factor 9 axis [153] and alleviate the inflammatory response [154]. Human amniotic fluid stem cell- or human amniotic epithelial cell-derived exosomes accelerate wound healing [155,156]. Moreover, human decidua MSC-derived exosomes can improve high-glucose senescent HDFs via intracellular miR-145-5p and miR-498 [157].

However, in the healing process, multiple internal cell types, including neutrophils, monocytes, macrophages, keratinocytes, fibroblasts, T cells, B cells, mast cells and endothelial cells are involved. These cells are actively associated with the production and regulation of various cytokines and growth factors [158], fight infection at the site of injury, form new tissues, and ultimately lead to wound closure [159]. MSC is ‘external assistance’, and these cells are ‘real participants’. EVs are also crucial in disease progression and anti-inflammatory repair, but the role of EVs is not fully understood and clarified. In the complex microenvironment of wound healing, skin tissue-EVs are more likely to express receptors for intercellular communication. The study developed a method of isolating keratinocyte-derived exosomes with genetically labeled GFP-reporter (Exo_κ_-GFP) from wound-edge tissue. Moreover, surface N-glycans of Exo_κ_-GFP were also characterized and glycan ions with high mannose were only detected in wound-edge Exo_κ_. Wound-edge KC exosomes revealed characteristic N-glycan ions with an abundance of low-base-pair RNA and were selectively engulfed by wound macrophages (ωmϕ) during granulation [87]. The Exo_κ_-GFP may be a regulator of macrophage trafficking and epithelial barrier post-injury. Additionally, skin tissue cell-EVs can act directly on homogeneous cells. These findings reveal that miR-125b is transferred via exosomes from young fibroblasts to old fibroblasts, contributing to the promotion of fibroblast migration and transition to counteract aging [160]. Growth factor-mediated cross-talk between keratinocytes and fibroblasts reveals the double paracrine signaling [104]. Some early research suggested that EVs have different characteristic molecular expression profiles due to their different sources and can act on different target cells with certain selectivity. For example, exosomes that express the transmembrane surface protein network tSPAN8-Alpha4 are more likely to enter endothelial cells and pancreatic cells [161]. Exosomes that express CD47 are less susceptible to uptake by monocytes [96]. Skin cell-EVs and MSC-EVs may have various molecular expression profiles and it may be speculated that specific skin cells-derived EVs could selectively function on target cells; however, the explicit difference between the two remains unclear and related research is lacking. Is there a unique advantage of the skin microenvironment for the self-repair effects of skin tissue cells? Recent studies have shown that EVs derived from ESCs exhibit more potent repair effects than other stromal cell exosomes do. Both ESC-Exo and FB-Exo promote diabetic wound closure, but ESC-Exo show better effects [162]. Various cells have special or common functions; for example, re-epithelialization is dependent on the ability of keratinocytes to form holo-clones [163]. Melanocytes and fibroblasts may contribute to re-pigmentation [164]. Cellular interactions at wound sites promote fibroblast attachment and produce a new connective tissue matrix and double paracrine interaction between keratinocytes and fibroblasts [165]. These findings suggest that EVs from skin tissues play unique roles in cutaneous wound repair. They can better reflect the cross-talk between cells in the wound healing environment and reveal the mechanism of injury repair. Further research will aid in the understanding of their contribution to wound healing in more detail and may explain the different clinical outcomes. Both skin cell-EVs and MSC-EVs possess great clinical application potential, but currently clinical trials mainly focus on MSC-EVs (Table 2). More potential and applications of skin-EVs require further research.

**Table 2:** Clinical trials with extracellular vesicles for cutaneous injury

**NCT number**	**Phase**	**Status**	**Condition**	**Source**	**Therapeutic**	**Reference**
05243368	Not yet applicable	Not yet recruiting	Diabetic foot	MSC		/
04173650	Phase I/II	Not yet recruiting	Dystrophic epidermolysis bullosa	MSC	AGLE 102	[165]
05078385	Phase I	Not yet recruiting	Burns	BM-MSC	AGLE-102	/
04134676	Phase I	Completed	Chronic ulcer	MSC	Conditioned medium	/
04235296	Phase I	Recruiting	Residual burn wound	MSC	Conditioned medium	/
03686449	Not yet applicable	Not yet recruiting	Post-burn raw area	Autologous KC ADSC-KC	Suspension	/
02565264	Early Phase I	Unknown	Ulcer	Plasma	Exosome	/
04134676	Phase I	Completed	Chronic ulcer	WJ-MSC	Conditioned medium	/
/	Preclinical		Skin lesions, inflammatory and autoimmune disorders	Not indicated	Exosome-based product	[166]
/	Preclinical	/	Skin lesions	ADSCs, placental MSCs, cord blood serum		[166]
/	/	/	Acne scars	Adipose tissue stem cell	Exosomes gel	[18]

*MSC* mesenchymal stem cells, *BM-MSC* bone marrow mesenchymal stem cells, *ADSCs* adipose-derived stem cells, *KC* keratinocyte, *WJ-MSC* Wharton's jelly mesenchymal stem cells

#### Complexity and risks of EV application in pathological conditions

The regenerative effects of EVs are at least in part attributed to the transfer of functional messages, which are highly dependent on specific conditions. The normal wound healing cascade is well coordinated and synchronized by growth factors, different MMPs, cytokines, inflammatory cells, keratinocytes, fibroblasts and endothelial cells. Chronic injury, such as diabetic wounds, exhibits a persistent inflammatory phase and is mainly characterized by the presence of chronic low-grade inflammation [166,167]. In pathological conditions, such as inflammation, aging and different living statuses, EVs may carry ‘undesired’ messages that contribute to the spread of diseases. Do EVs secreted by specific skin tissue cells consistently exhibit the supporting effect?

Macrophages are pivotal contributors to healing; however, inflammatory cells are also a double-edged sword, and EVs as a medium of communication may have the opposite effect in the context of persistent inflammatory infiltration. Research has indicated that defective MV secretion/function may cause aberrant wound healing and chronic wounds that fail to re-epithelialize [168]. Evidence indicates that mast cell exosomes can produce cytosolic PLA2 and contribute to a CD1a-reactive T cell response in psoriasis, a chronic inflammatory skin disease [86]. The study discovered that keratinocyte exosomes activate neutrophils and enhance skin inflammation [169]. Similarly, neutrophil exosomes can also stimulate keratinocytes and increase skin autoinflammation in generalized pustular psoriasis [170]. This reveals that exosomes not only work as a means of keratinocyte-neutrophil communication but also show detrimental effects. In allergic inflammation, exosomes mediate cellular interactions by regulating the ROS levels [171,172].

Aging is accompanied by chronic low-grade systemic inflammation [173]. Senescent cells accumulate in the skin during aging and alter the skin’s microenvironment [174,175]. Research has proved that fibroblasts from aged skin exhibit a senescence-associated secretory phenotype rich in pro-inflammatory cytokines, chemokines and proteases, which can have a detrimental impact on nearby cells and the local tissue environment [105]. Choi et al. also demonstrated that EVs derived from senescent fibroblasts attenuated the dermal effect on keratinocyte differentiation [176]. EVs in the aging microenvironment may have detrimental effects on downstream targets at the levels of immunity, inflammation, gene expression, and metabolism [75]. Additionally, keratinocytes are divided into differentiated (diff-K) and undifferentiated keratinocytes (undiff-K). The EVs produced by them also differ in their contents and effects. The study found exosomes containing 14-3-3s from diff-K had a higher MMP-1 stimulatory effect in fibroblasts than exosomes isolated from undiff-K [177].

#### Intrinsic challenges of EVs

At present, EVs derived from stem cells have been developed into products and are gradually being applied from the bench to the bedside. For example, exosome repair fluid is used for scars or in the form of dressings. Although exosomes have broad prospects in the field of skin repair and cosmetic medicine, there are still several challenges in their clinical application. For instance, a lack of appropriate methodology, inadequate cell number availability, insufficient targeting and poor stability in circulation [178–180].

#### Technical challenges

Currently, we do not yet have the tools needed to properly study EVs. The heterogeneity of sEVs and the presence of nonvesicular extracellular substances have led to debate about the contents and functional properties of exosomes [181]. Several commonly used EV purification methods have been developed, but all have limitations, such as producing EVs with different yields and purities [18,182]. To improve the purity of separation, the first problem to be addressed is to clarify the classification of EVs and the specific differences between sEV, other EVs, and nonvesicular in physical and chemical aspects, such as size, density, surface protein, sugar, lipid components and surface charge [180]. High-resolution density gradient fractionation and direct immunoaffinity capture were employed to precisely characterize the RNA, DNA, and protein constituents of exosomes and other nonvesicular materials. They found that extracellular RNA, RNA-binding proteins, and other cellular proteins were differentially expressed in exosomes and nonvesicle compartments [183], allowing for more precise determination of the molecular composition of classical exosomes.

#### Inherent defects in natural EVs

To overcome the inherent shortcomings of EVs, researchers have enhanced their capabilities by designing or generating biomimetic EVs. Wang et al. developed an injectable, self-healing and antibacterial polypeptide-based FHE hydrogel, which is named from its composition of pluronic F127 (F127), oxidative hyaluronic acid (OHA), and ε-poly-l-lysine, polypeptide (EPL) with exosomes to accelerate diabetic wound healing [184]. Shen et al. designed bilayered thiolated alginate/polyethylene glycol diacrylate hydrogels for the sequential release of sEVs to achieve rapid and scarless wound healing [185]. The hydrogel itself provided a moist environment, which was conducive to debridement, and the hydrogel-loaded EVs enhanced their stability *in vivo* and played a role in sustained release [186]. Cutaneous injury is often accompanied by infection, so the combination of antibacterial materials and EVs is also a research hotspot, to play a dual role. Dressing loaded-exosomes and AgNPs have antimicrobial activity and promote wound healing [187]. Similarly, exosome-laden oxygen-releasing antioxidant and antibacterial cryogel wound dressing OxOBand could support diabetic wound closure [188]. Membrane-targeting modification of EVs is also a strategy. Su et al. demonstrated that PD-L1 overexpression in exosomes could be specifically bound to PD-1 on the T cell surface and suppressed T cell activation to facilitate tissue repair [189]. Immunosuppression of excessive or persistent inflammation during wound healing is another effective approach. To enhance the organ-targeting ability of EVs, Li et al. fabricated iron oxide nanoparticle (NP)-labeled exosomes (Exo + NPs) and found that they were readily internalized by HUVECs [190]. Whether natural or engineered, EVs show a strong ability to repair damage and engineered EVs constitute a new strategy and may soon be developed as smart drugs for clinical treatment.

#### Clinical translational opportunities

To achieve the application of EVs from the bench to the bedside, research has focused on the basic biology of EVs, including their biogenesis, dynamics in extracellular space, transit and uptake by target cells. To completely capitalize on their potential, a better understanding of the cellular and molecular mechanisms that govern the many observed functions of EVs is required. Numerous issues need to be acknowledged; for example, how do EVs interact with different matrices? How are EVs maintained in (or cleared from) biological fluid? How do they cross the biological barriers? How are they determined in specific tissues? How are they taken up into cells, and how are cargo functionally delivered? The dose and safety should be evaluated in clinical applications. The systemic application of EVs may induce apoptosis in some cells, including immune regulation [191]. Generally, the wound is used locally, but whether systemic or local, EVs are easily cleared rapidly [192]. Topical EVs are, in the form of liquid dressings, sprays or solid plasters. Different forms of EVs may affect the state of EVs and have different effects. Additionally, the injection dose of EVs must be considered, as different doses can produce various biological effects. When EVs are applied for the treatment of neurodegenerative diseases, it has been shown *in vitro* that low doses of EVs have neuroprotective effects, whereas high doses may be harmful to neurons [193]. EVs may exert similar effects on wound repair. Moreover, injection of EVs into the body may induce an immune response, and there are differences in individual responses [194]. EVs have exhibited superior therapeutic effects in regenerative medicine; however, most applications are still in the experimental stage. A great deal of clinical validation is needed for the regulatory approval of EV-based diagnostics and therapeutics. An easier, efficient, safe and high-yield method for EVs production must be developed to provide better EVs for future clinical use. In summary, further studies on EVs and their gradual clinical trials in skin injury repair have broad application prospects.

## Conclusions

This review discusses the effects of EVs derived from specific epidermal cells, such as dermis and inflammatory cells, on cutaneous repair and regeneration. EVs have high stability, biocompatibility, low toxicity, and immunogenicity. Therefore, they are potential candidates for novel cell-free based therapies for different diseases, including skin tissue repair. While not intended to substitute completely for classical forms of intercellular communication (hormones, chemokines, cytokines and mitogens), EVs provide a local environment potentially and modify the physiology and functional activities of cells. The study of special skin tissue cell-derived EVs in wound healing will be beneficial for revealing the dynamic process of wound healing and the specific mechanism of cell function. Taken together, this study provides novel insights into EVs derived from specific skin tissue cells and the importance of cross-talk between different skin cell types in wound healing. However, many open questions remain regarding the signaling molecules and uptake of EVs that participate in the EVs-induced cell response. Little is known about the dynamics of EVs in wound healing. Mechanistically, it would be interesting to further elucidate how cells interact with and influence each other. Therefore, further research is required to clarify the regulation of EVs during the wound healing process and to translate these results to clinical settings.

## Abbreviations

BM: Bone marrow; DFs-EVs: Dermal fibroblasts-derived EVs; diff-K: Differentiated keratinocytes; DPCs: Dermal papilla cells; DPC-EVs: Dermal papilla cells-derived EVs; ECM: Extracellular matrix; EMT: Epithelial–mesenchymal transition; EPC-EVs: Endothelial progenitor cells-derived EVs; ESC-EVs: Epidermal stem cells-derived EVs; ESC-Exo: Epidermal stem cells-derived exosomes; ESCRT: Endosomal sorting complex required for transport; EV: extracellular vesicle; FB-Exo: Fibroblast exosomes; HDFs: Human dermal fibroblasts; HDFs-EVs: Human dermal fibroblasts-derived EVs; HUVECs: Human umbilical vein endothelial cells; ILVs: Intraluminal vesicles; iPSCs: Induced pluripotent stem cells; KC-EVs: Keratinocyte-derived EVs; MAC-EVs: Macrophage-derived EVs; MITF: Microphthalmia-associated transcription factor; MMP: Matrix metalloproteinase; MSC-EVs: Mesenchymal stem cell-derived EVs; MVs: Microvesicles; MVBs: Multivesicular bodies; NETs: Neutrophil extracellular traps; ORS: Outer root sheath; ROS: Reactive oxygen species; sEVs: Small EVs; undiff-K: Undifferentiated keratinocytes; VEGF: Vascular endothelial growth factor; FHE: Pluronic F127 (F127); OHA: oxidative hyaluronic acid; EPL: polypeptide ε-poly-l-lysine

## References

[1] Singer AJ, Clark RA. Cutaneous wound healing. N Engl J Med. 1999;341:738–46. 10471461 10.1056/NEJM199909023411006

[2] Marti-Carvajal AJ, Gluud C, Nicola S, Simancas-Racines D, Reveiz L, Oliva P, et al. Growth factors for treating diabetic foot ulcers. Cochrane Database Syst Rev. 2015;2015:CD008548. 10.1002/14651858.CD008548.pub2 PMC8665376 26509249

[3] Raposo G, Stoorvogel W. Extracellular vesicles: exosomes, microvesicles, and friends. J Cell Biol. 2013;200:373–83. 23420871 10.1083/jcb.201211138 PMC3575529

[4] Rodrigues M, Kosaric N, Bonham CA, Gurtner GC. Wound healing: a cellular perspective. Physiol Rev. 2019;99:665–706. 30475656 10.1152/physrev.00067.2017 PMC6442927

[5] Sun BK, Siprashvili Z, Khavari PA. Advances in skin grafting and treatment of cutaneous wounds. Science. 2014;346:941–5. 25414301 10.1126/science.1253836

[6] Broughton GN, Janis JE, Attinger CE. Wound healing: an overview. Plast Reconstr Surg. 2006;117:1e–32e. 16801750 10.1097/01.prs.0000222562.60260.f9

[7] Minutti CM, Knipper JA, Allen JE, Zaiss DM. Tissue-specific contribution of macrophages to wound healing. Semin Cell Dev Biol. 2017;61:3–11. 27521521 10.1016/j.semcdb.2016.08.006

[8] Huang P, Bi J, Owen GR, Chen W, Rokka A, Koivisto L, et al. Keratinocyte microvesicles regulate the expression of multiple genes in dermal fibroblasts. J Invest Dermatol. 2015;135:3051–9. 26288358 10.1038/jid.2015.320

[9] Marconi GD, Fonticoli L, Rajan TS, Pierdomenico SD, Trubiani O, Pizzicannella J, et al. Epithelial-mesenchymal transition (EMT): the type-2 EMT in wound healing, tissue regeneration and organ fibrosis. Cells. 2021;10:1587. 10.3390/cells10071587 PMC8307661 34201858

[10] Chitturi RT, Balasubramaniam AM, Parameswar RA, Kesavan G, Haris KT, Mohideen K. The role of myofibroblasts in wound healing, contraction and its clinical implications in cleft palate repair. J Int Oral Health. 2015;7:75–80. PMC4385733 25878485

[11] Marconi GD, Fonticoli L, Rajan TS, Lanuti P, Della RY, Pierdomenico SD, et al. Transforming growth factor-beta1 and human gingival fibroblast-to-myofibroblast differentiation: molecular and morphological modifications. Front Physiol. 2021;12:676512. 34093237 10.3389/fphys.2021.676512 PMC8176099

[12] Cocozza F, Grisard E, Martin-Jaular L, Mathieu M, Théry C. SnapShot: Extracellular vesicles. Cell. 2020;182:262. 32649878 10.1016/j.cell.2020.04.054

[13] Yanez-Mo M, Siljander PR, Andreu Z, Zavec AB, Borras FE, Buzas EI, et al. Biological properties of extracellular vesicles and their physiological functions. *J Extracell Vesicles*. 2015;4:27066. 25979354 10.3402/jev.v4.27066 PMC4433489

[14] Ratajczak J, Miekus K, Kucia M, Zhang J, Reca R, Dvorak P, et al. Embryonic stem cell-derived microvesicles reprogram hematopoietic progenitors: evidence for horizontal transfer of mRNA and protein delivery. Leukemia. 2006;20:847–56. 16453000 10.1038/sj.leu.2404132

[15] Gatti S, Bruno S, Deregibus MC, Sordi A, Cantaluppi V, Tetta C, et al. Microvesicles derived from human adult mesenchymal stem cells protect against ischaemia-reperfusion-induced acute and chronic kidney injury. Nephrol Dial Transplant. 2011;26:1474–83. 21324974 10.1093/ndt/gfr015

[16] Lindenbergh M, Stoorvogel W. Antigen presentation by extracellular vesicles from professional antigen-presenting cells. Annu Rev Immunol. 2018;36:435–59. 29400984 10.1146/annurev-immunol-041015-055700

[17] Del CI, Shrimpton CN, Thiagarajan P, Lopez JA. Tissue-factor-bearing microvesicles arise from lipid rafts and fuse with activated platelets to initiate coagulation. Blood. 2005;106:1604–11. 15741221 10.1182/blood-2004-03-1095

[18] Thery C, Witwer KW, Aikawa E, Alcaraz MJ, Anderson JD, Andriantsitohaina R, et al. Minimal information for studies of extracellular vesicles 2018 (MISEV2018): a position statement of the International Society for Extracellular Vesicles and update of the MISEV2014 guidelines. J Extracell Vesicles. 2018;7:1535750. 30637094 10.1080/20013078.2018.1535750 PMC6322352

[19] Shao H, Im H, Castro CM, Breakefield X, Weissleder R, Lee H. New technologies for analysis of extracellular vesicles. Chem Rev. 2018;118:1917–50. 29384376 10.1021/acs.chemrev.7b00534 PMC6029891

[20] Gurunathan S, Kang MH, Jeyaraj M, Qasim M, Kim JH. Review of the isolation, characterization, biological function, and multifarious therapeutic approaches of exosomes. Cells. 2019;8:307. 10.3390/cells8040307 PMC6523673 30987213

[21] Lee Y, El AS, Wood MJ. Exosomes and microvesicles: extracellular vesicles for genetic information transfer and gene therapy. Hum Mol Genet. 2012;21:R125–34. 22872698 10.1093/hmg/dds317

[22] Wollert T, Hurley JH. Molecular mechanism of multivesicular body biogenesis by ESCRT complexes. Nature. 2010;464:864–9. 20305637 10.1038/nature08849 PMC2851844

[23] Colombo M, Raposo G, Thery C. Biogenesis, secretion, and intercellular interactions of exosomes and other extracellular vesicles. Annu Rev Cell Dev Biol. 2014;30:255–89. 25288114 10.1146/annurev-cellbio-101512-122326

[24] Verderio C, Gabrielli M, Giussani P. Role of sphingolipids in the biogenesis and biological activity of extracellular vesicles. J Lipid Res. 2018;59:1325–40. 29853528 10.1194/jlr.R083915 PMC6071771

[25] Johnstone RM, Adam M, Hammond JR, Orr L, Turbide C. Vesicle formation during reticulocyte maturation. Association of plasma membrane activities with released vesicles (exosomes). J Biol Chem. 1987;262:9412–20. 3597417

[26] Zhao D, Tao W, Li S, Chen Y, Sun Y, He Z, et al. Apoptotic body-mediated intercellular delivery for enhanced drug penetration and whole tumor destruction. Sci Adv. 2021;7:eabg0880. 10.1126/sciadv.abg0880 PMC8051881 33863733

[27] Hugel B, Martinez MC, Kunzelmann C, Freyssinet JM. Membrane microparticles: two sides of the coin. Physiology (Bethesda). 2005;20:22–7. 15653836 10.1152/physiol.00029.2004

[28] Li S, Li Y, Chen B, Zhao J, Yu S, Tang Y, et al. exoRBase: a database of circRNA, lncRNA and mRNA in human blood exosomes. Nucleic Acids Res. 2018;46:D106–12. 30053265 10.1093/nar/gkx891 PMC5753357

[29] He C, Zheng S, Luo Y, Wang B. Exosome theranostics: biology and translational medicine. Theranostics. 2018;8:237–55. 29290805 10.7150/thno.21945 PMC5743472

[30] Thakur BK, Zhang H, Becker A, Matei I, Huang Y, Costa-Silva B, et al. Double-stranded DNA in exosomes: a novel biomarker in cancer detection. Cell Res. 2014;24:766–9. 24710597 10.1038/cr.2014.44 PMC4042169

[31] Sansone P, Savini C, Kurelac I, Chang Q, Amato LB, Strillacci A, et al. Packaging and transfer of mitochondrial DNA via exosomes regulate escape from dormancy in hormonal therapy-resistant breast cancer. Proc Natl Acad Sci U S A. 2017;114:E9066–75. 29073103 10.1073/pnas.1704862114 PMC5664494

[32] Record M, Silvente-Poirot S, Poirot M, Wakelam M. Extracellular vesicles: lipids as key components of their biogenesis and functions. J Lipid Res. 2018;59:1316–24. 29764923 10.1194/jlr.E086173 PMC6071772

[33] Colombo M, Moita C, van Niel G, Kowal J, Vigneron J, Benaroch P, et al. Analysis of ESCRT functions in exosome biogenesis, composition and secretion highlights the heterogeneity of extracellular vesicles. J Cell Sci. 2013;126:5553–65. 24105262 10.1242/jcs.128868

[34] Baietti MF, Zhang Z, Mortier E, Melchior A, Degeest G, Geeraerts A, et al. Syndecan-syntenin-ALIX regulates the biogenesis of exosomes. Nat Cell Biol. 2012;14:677–85. 22660413 10.1038/ncb2502

[35] van Niel G, Charrin S, Simoes S, Romao M, Rochin L, Saftig P, et al. The tetraspanin CD63 regulates ESCRT-independent and -dependent endosomal sorting during melanogenesis. Dev Cell. 2011;21:708–21. 21962903 10.1016/j.devcel.2011.08.019 PMC3199340

[36] Thom SR, Bhopale VM, Yu K, Huang W, Kane MA, Margolis DJ. Neutrophil microparticle production and inflammasome activation by hyperglycemia due to cytoskeletal instability. J Biol Chem. 2017;292:18312–24. 28972154 10.1074/jbc.M117.802629 PMC5672053

[37] Wehman AM, Poggioli C, Schweinsberg P, Grant BD, Nance J. The P4-ATPase TAT-5 inhibits the budding of extracellular vesicles in C. elegans embryos. Curr Biol. 2011;21:1951–9. 22100064 10.1016/j.cub.2011.10.040 PMC3237752

[38] Anand S, Foot N, Ang CS, Gembus KM, Keerthikumar S, Adda CG, et al. Arrestin-domain containing protein 1 (Arrdc1) regulates the protein cargo and release of extracellular vesicles. Proteomics. 2018;18:e1800266. 30035390 10.1002/pmic.201800266

[39] van Niel G, Carter D, Clayton A, Lambert DW, Raposo G, Vader P. Challenges and directions in studying cell-cell communication by extracellular vesicles. Nat Rev Mol Cell Biol. 2022;23:369–82. 35260831 10.1038/s41580-022-00460-3

[40] Campanella C, Caruso BC, Logozzi M, Marino GA, Mizzoni D, Cappello F, et al. On the choice of the extracellular vesicles for therapeutic purposes. Int J Mol Sci. 2019;20:236. 10.3390/ijms20020236 PMC6359369 30634425

[41] Ding Y, Li Y, Sun Z, Han X, Chen Y, Ge Y, et al. Cell-derived extracellular vesicles and membranes for tissue repair. J Nanobiotechnology. 2021;19:368. 34789267 10.1186/s12951-021-01113-x PMC8600774

[42] Rajan TS, Diomede F, Bramanti P, Trubiani O, Mazzon E. Conditioned medium from human gingival mesenchymal stem cells protects motor-neuron-like NSC-34 cells against scratch-injury-induced cell death. Int J Immunopathol Pharmacol. 2017;30:383–94. 29140156 10.1177/0394632017740976 PMC5806806

[43] Silvestro S, Chiricosta L, Gugliandolo A, Pizzicannella J, Diomede F, Bramanti P, et al. Extracellular vesicles derived from human gingival mesenchymal stem cells: a transcriptomic analysis. Genes (Basel). 2020;11:118. 10.3390/genes11020118 PMC7073771 31973135

[44] Rani S, Ritter T. The exosome – a naturally secreted nanoparticle and its application to wound healing. Adv Mater. 2016;28:5542–52. 26678528 10.1002/adma.201504009

[45] Wu P, Zhang B, Shi H, Qian H, Xu W. MSC-exosome: a novel cell-free therapy for cutaneous regeneration. Cytotherapy. 2018;20:291–301. 29434006 10.1016/j.jcyt.2017.11.002

[46] Hu Q, Lyon CJ, Fletcher JK, Tang W, Wan M, Hu TY. Extracellular vesicle activities regulating macrophage- and tissue-mediated injury and repair responses. Acta Pharm Sin B. 2021;11:1493–512. 34221864 10.1016/j.apsb.2020.12.014 PMC8245807

[47] Lombardo G, Gili M, Grange C, Cavallari C, Dentelli P, Togliatto G, et al. IL-3R-alpha blockade inhibits tumor endothelial cell-derived extracellular vesicle (EV)-mediated vessel formation by targeting the beta-catenin pathway. Oncogene. 2018;37:1175–91. 29238040 10.1038/s41388-017-0034-x PMC5861089

[48] Adamo A, Brandi J, Caligola S, Delfino P, Bazzoni R, Carusone R, et al. Extracellular vesicles mediate mesenchymal stromal cell-dependent regulation of B cell PI3K-AKT signaling pathway and actin cytoskeleton. Front Immunol. 2019;10:446. 30915084 10.3389/fimmu.2019.00446 PMC6423067

[49] Maggio S, Ceccaroli P, Polidori E, Cioccoloni A, Stocchi V, Guescini M. Signal exchange through extracellular vesicles in neuromuscular junction establishment and maintenance: from physiology to pathology. Int J Mol Sci. 2019;20:2804. 10.3390/ijms20112804 PMC6600513 31181747

[50] Han M, Cao Y, Xue H, Chu X, Li T, Xin D, et al. Neuroprotective effect of mesenchymal stromal cell-derived extracellular vesicles against cerebral ischemia-reperfusion-induced neural functional injury: a pivotal role for AMPK and JAK2/STAT3/NF-kappaB signaling pathway modulation. Drug Des Devel Ther. 2020;14:2865–76. 10.2147/DDDT.S248892 PMC7381771 32764885

[51] Zhou Y, Xiao Y. The development of extracellular vesicle-integrated biomaterials for bone regeneration. Adv Exp Med Biol. 2020;1250:97–108. 32601940 10.1007/978-981-15-3262-7_7

[52] Huang J, Xiong J, Yang L, Zhang J, Sun S, Liang Y. Cell-free exosome-laden scaffolds for tissue repair. Nanoscale. 2021;13:8740–50. 33969373 10.1039/d1nr01314a

[53] Kim G, Lee Y, Ha J, Han S, Lee M. Engineering exosomes for pulmonary delivery of peptides and drugs to inflammatory lung cells by inhalation. J Control Release. 2021;330:684–95. 33388343 10.1016/j.jconrel.2020.12.053

[54] Tian T, Zhang HX, He CP, Fan S, Zhu YL, Qi C, et al. Surface functionalized exosomes as targeted drug delivery vehicles for cerebral ischemia therapy. Biomaterials. 2018;150:137–49. 29040874 10.1016/j.biomaterials.2017.10.012

[55] Zhuang X, Xiang X, Grizzle W, Sun D, Zhang S, Axtell RC, et al. Treatment of brain inflammatory diseases by delivering exosome encapsulated anti-inflammatory drugs from the nasal region to the brain. Mol Ther. 2011;19:1769–79. 21915101 10.1038/mt.2011.164 PMC3188748

[56] Bjorge IM, Kim SY, Mano JF, Kalionis B, Chrzanowski W. Extracellular vesicles, exosomes and shedding vesicles in regenerative medicine – a new paradigm for tissue repair. Biomater Sci. 2017;6:60–78. 29184934 10.1039/c7bm00479f

[57] Haney MJ, Klyachko NL, Zhao Y, Gupta R, Plotnikova EG, He Z, et al. Exosomes as drug delivery vehicles for Parkinson's disease therapy. J Control Release. 2015;207:18–30. 25836593 10.1016/j.jconrel.2015.03.033 PMC4430381

[58] Nakao Y, Fukuda T, Zhang Q, Sanui T, Shinjo T, Kou X, et al. Exosomes from TNF-alpha-treated human gingiva-derived MSCs enhance M2 macrophage polarization and inhibit periodontal bone loss. Acta Biomater. 2021;122:306–24. 33359765 10.1016/j.actbio.2020.12.046 PMC7897289

[59] Dalirfardouei R, Gholoobi A, Vahabian M, Mahdipour E, Afzaljavan F. Therapeutic role of extracellular vesicles derived from stem cells in cutaneous wound models: a systematic review. Life Sci. 2021;273:119271. 33652035 10.1016/j.lfs.2021.119271

[60] Vu NB, Nguyen HT, Palumbo R, Pellicano R, Fagoonee S, Pham PV. Stem cell-derived exosomes for wound healing: current status and promising directions. Minerva Med. 2021;112:384–400. 33263376 10.23736/S0026-4806.20.07205-5

[61] An Y, Lin S, Tan X, Zhu S, Nie F, Zhen Y, et al. Exosomes from adipose-derived stem cells and application to skin wound healing. Cell Prolif. 2021;54:e12993. 33458899 10.1111/cpr.12993 PMC7941238

[62] Wei F, Wang A, Wang Q, Han W, Rong R, Wang L, et al. Plasma endothelial cells-derived extracellular vesicles promote wound healing in diabetes through YAP and the PI3K/Akt/mTOR pathway. Aging (Albany NY). 2020;12:12002–18. 32570219 10.18632/aging.103366 PMC7343472

[63] Ye M, Ni Q, Qi H, Qian X, Chen J, Guo X, et al. Exosomes derived from human induced pluripotent stem cells-endothelia cells promotes postnatal angiogenesis in mice bearing ischemic limbs. Int J Biol Sci. 2019;15:158–68. 30662356 10.7150/ijbs.28392 PMC6329927

[64] Kim MH, Chung C, Oh MH, Jun JH, Ko Y, Lee JH. Extracellular vesicles from a three-dimensional culture of perivascular cells accelerate skin wound healing in a rat. Aesthet Plast Surg. 2021;45:2437–46. 10.1007/s00266-021-02254-y 33821312

[65] Jansen EE, Braun A, Jansen P, Hartmann M. Platelet-therapeutics to improve tissue regeneration and wound healing-physiological background and methods of preparation. Biomedicine. 2021;9:869. 10.3390/biomedicines9080869 PMC8389548 34440073

[66] Cretoiu D, Gherghiceanu M, Hummel E, Zimmermann H, Simionescu O, Popescu LM. FIB-SEM tomography of human skin telocytes and their extracellular vesicles. J Cell Mol Med. 2015;19:714–22. 25823591 10.1111/jcmm.12578 PMC4395186

[67] Nakamura K, Okuyama R. Immunotherapy for advanced melanoma: current knowledge and future directions. J Dermatol Sci. 2016;83:87–94. 27302423 10.1016/j.jdermsci.2016.05.009

[68] Xie J, Yao B, Han Y, Huang S, Fu X. Skin appendage-derived stem cells: cell biology and potential for wound repair. Burns Trauma. 2016;4:38. 27800498 10.1186/s41038-016-0064-6 PMC5082359

[69] Jang Y, Lee AY, Chang SH, Jeong SH, Park KH, Paik MK, et al. Trifloxystrobin induces tumor necrosis factor-related apoptosis-inducing ligand (TRAIL)-mediated apoptosis in HaCaT, human keratinocyte cells. Drug Chem Toxicol. 2017;40:67–73. 27149887 10.1080/01480545.2016.1174871

[70] Lo CA, Stahl PD, Raposo G. Extracellular vesicles shuffling intercellular messages: for good or for bad. Curr Opin Cell Biol. 2015;35:69–77. 26001269 10.1016/j.ceb.2015.04.013

[71] Merjaneh M, Langlois A, Larochelle S, Cloutier CB, Ricard-Blum S, Moulin VJ. Pro-angiogenic capacities of microvesicles produced by skin wound myofibroblasts. Angiogenesis. 2017;20:385–98. 28391377 10.1007/s10456-017-9554-9

[72] Waster P, Eriksson I, Vainikka L, Rosdahl I, Ollinger K. Extracellular vesicles are transferred from melanocytes to keratinocytes after UVA irradiation. Sci Rep. 2016;6:27890. 27293048 10.1038/srep27890 PMC4904274

[73] Ter Horst B, Chouhan G, Moiemen NS, Grover LM. Advances in keratinocyte delivery in burn wound care. Adv Drug Deliv Rev. 2018;123:18–32. 28668483 10.1016/j.addr.2017.06.012 PMC5764224

[74] Chavez-Munoz C, Morse J, Kilani R, Ghahary A. Primary human keratinocytes externalize stratifin protein via exosomes. J Cell Biochem. 2008;104:2165–73. 18452139 10.1002/jcb.21774

[75] Chavez-Munoz C, Kilani RT, Ghahary A. Profile of exosomes related proteins released by differentiated and undifferentiated human keratinocytes. J Cell Physiol. 2009;221:221–31. 19530224 10.1002/jcp.21847

[76] Than U, Leavesley DI, Parker TJ. Characteristics and roles of extracellular vesicles released by epidermal keratinocytes. J Eur Acad Dermatol Venereol. 2019;33:2264–72. 31403744 10.1111/jdv.15859

[77] Lo CA, Delevoye C, Gilles-Marsens F, Loew D, Dingli F, Guere C, et al. Exosomes released by keratinocytes modulate melanocyte pigmentation. Nat Commun. 2015;6:7506. 26103923 10.1038/ncomms8506 PMC4491833

[78] Seiberg M . Keratinocyte-melanocyte interactions during melanosome transfer. Pigment Cell Res. 2001;14:236–42. 11549105 10.1034/j.1600-0749.2001.140402.x

[79] Liu Y, Xue L, Gao H, Chang L, Yu X, Zhu Z, et al. Exosomal miRNA derived from keratinocytes regulates pigmentation in melanocytes. J Dermatol Sci. 2019;93:159–67. 30904353 10.1016/j.jdermsci.2019.02.001

[80] Liu L, Awoyemi AA, Fahy KE, Thapa P, Borchers C, Wu BY, et al. Keratinocyte-derived microvesicle particles mediate ultraviolet B radiation-induced systemic immunosuppression. J Clin Invest. 2021;131:e144963. 10.1172/JCI144963 PMC8121517 33830943

[81] Papayannakos CJ, DeVoti JA, Israr M, Alsudani H, Bonagura V, Steinberg BM. Extracellular vesicles produced by primary human keratinocytes in response to TLR agonists induce stimulus-specific responses in antigen-presenting cells. Cell Signal. 2021;83:109994. 33781846 10.1016/j.cellsig.2021.109994 PMC8091864

[82] Takada Y, Ye X, Simon S. The integrins. Genome Biol. 2007;8:215. 17543136 10.1186/gb-2007-8-5-215 PMC1929136

[83] Li Q, Zhao H, Chen W, Huang P, Bi J. Human keratinocyte-derived microvesicle miRNA-21 promotes skin wound healing in diabetic rats through facilitating fibroblast function and angiogenesis. Int J Biochem Cell Biol. 2019;114:105570. 31302227 10.1016/j.biocel.2019.105570

[84] Nasiri G, Azarpira N, Alizadeh A, Goshtasbi S, Tayebi L. Shedding light on the role of keratinocyte-derived extracellular vesicles on skin-homing cells. Stem Cell Res Ther. 2020;11:421. 32993791 10.1186/s13287-020-01929-8 PMC7523352

[85] Glady A, Vandebroek A, Yasui M. Human keratinocyte-derived extracellular vesicles activate the MAPKinase pathway and promote cell migration and proliferation in vitro. Inflamm Regen. 2021;41:4. 33526070 10.1186/s41232-021-00154-x PMC7852286

[86] Jiang M, Fang H, Shao S, Dang E, Zhang J, Qiao P, et al. Keratinocyte exosomes activate neutrophils and enhance skin inflammation in psoriasis. FASEB J. 2019;33:13241–53. 31539277 10.1096/fj.201900642R

[87] Zhou X, Brown BA, Siegel AP, El MM, Zeng X, Song W, et al. Exosome-mediated crosstalk between keratinocytes and macrophages in cutaneous wound healing. ACS Nano. 2020;14:12732–48. 32931251 10.1021/acsnano.0c03064 PMC7970718

[88] Piipponen M, Li D, Landen NX. The immune functions of keratinocytes in skin wound healing. Int J Mol Sci. 2020;21:8790. 10.3390/ijms21228790 PMC7699912 33233704

[89] Jiang M, Fang H, Dang E, Zhang J, Qiao P, Yu C, et al. Small extracellular vesicles containing miR-381-3p from keratinocytes promote T helper type 1 and T helper type 17 polarization in psoriasis. J Invest Dermatol. 2021;141:563–74. 32712160 10.1016/j.jid.2020.07.009

[90] Ogawa M, Udono M, Teruya K, Uehara N, Katakura Y. Exosomes derived from fisetin-treated keratinocytes mediate hair growth promotion. Nutrients. 2021;13:2087. 10.3390/nu13062087 PMC8234638 34207142

[91] Yang R, Liu F, Wang J, Chen X, Xie J, Xiong K. Epidermal stem cells in wound healing and their clinical applications. Stem Cell Res Ther. 2019;10:229. 31358069 10.1186/s13287-019-1312-z PMC6664527

[92] Blanpain C, Fuchs E. Epidermal stem cells of the skin. Annu Rev Cell Dev Biol. 2006;22:339–73. 16824012 10.1146/annurev.cellbio.22.010305.104357 PMC2405915

[93] Wang ZL, He RZ, Tu B, He JS, Cao X, Xia HS, et al. Drilling combined with adipose-derived stem cells and bone morphogenetic protein-2 to treat femoral head epiphyseal necrosis in juvenile rabbits. Curr Med Sci. 2018;38:277–88. 30074186 10.1007/s11596-018-1876-3

[94] Leng L, Ma J, Lv L, Wang W, Gao D, Zhu Y, et al. Both Wnt signaling and epidermal stem cell-derived extracellular vesicles are involved in epidermal cell growth. Stem Cell Res Ther. 2020;11:415. 32967725 10.1186/s13287-020-01933-y PMC7510321

[95] Duan M, Zhang Y, Zhang H, Meng Y, Qian M, Zhang G. Epidermal stem cell-derived exosomes promote skin regeneration by downregulating transforming growth factor-beta1 in wound healing. Stem Cell Res Ther. 2020;11:452. 33097078 10.1186/s13287-020-01971-6 PMC7584097

[96] Wang P, Theocharidis G, Vlachos IS, Kounas K, Lobao A, Shu B, et al. Exosomes derived from epidermal stem cells improve diabetic wound healing. J Invest Dermatol. 2022;S0022–202X:00119–1. 10.1016/j.jid.2022.01.030 35181300

[97] Stunova A, Vistejnova L. Dermal fibroblasts – a heterogeneous population with regulatory function in wound healing. Cytokine Growth Factor Rev. 2018;39:137–50. 29395658 10.1016/j.cytogfr.2018.01.003

[98] Ichim TE, O'Heeron P, Kesari S. Fibroblasts as a practical alternative to mesenchymal stem cells. J Transl Med. 2018;16:212. 30053821 10.1186/s12967-018-1536-1 PMC6064181

[99] Rognoni E, Pisco AO, Hiratsuka T, Sipila KH, Belmonte JM, Mobasseri SA, et al. Fibroblast state switching orchestrates dermal maturation and wound healing. Mol Syst Biol. 2018;14:e8174. 30158243 10.15252/msb.20178174 PMC6113774

[100] Hwang YP, Oh KN, Yun HJ, Jeong HG. The flavonoids apigenin and luteolin suppress ultraviolet A-induced matrix metalloproteinase-1 expression via MAPKs and AP-1-dependent signaling in HaCaT cells. J Dermatol Sci. 2011;61:23–31. 21112745 10.1016/j.jdermsci.2010.10.016

[101] Han X, Wu P, Li L, Sahal HM, Ji C, Zhang J, et al. Exosomes derived from autologous dermal fibroblasts promote diabetic cutaneous wound healing through the Akt/beta-catenin pathway. Cell Cycle. 2021;20:616–29. 33685347 10.1080/15384101.2021.1894813 PMC8018430

[102] Deng M, Yu TZ, Li D, Wang X, Zhou G, Liu W, et al. Human umbilical cord mesenchymal stem cell-derived and dermal fibroblast-derived extracellular vesicles protect dermal fibroblasts from ultraviolet radiation-induced photoaging in vitro. Photochem Photobiol Sci. 2020;19:406–14. 32125331 10.1039/c9pp00421a

[103] Hu S, Li Z, Cores J, Huang K, Su T, Dinh PU, et al. Needle-free injection of exosomes derived from human dermal fibroblast spheroids ameliorates skin photoaging. ACS Nano. 2019;13:11273–82. 31449388 10.1021/acsnano.9b04384 PMC7032013

[104] van Niel G, D'Angelo G, Raposo G. Shedding light on the cell biology of extracellular vesicles. Nat Rev Mol Cell Biol. 2018;19:213–28. 29339798 10.1038/nrm.2017.125

[105] Choi EJ, Kil IS, Cho EG. Extracellular vesicles derived from senescent fibroblasts attenuate the dermal effect on keratinocyte differentiation. Int J Mol Sci. 2020;21:1022. 10.3390/ijms21031022 PMC7037765 32033114

[106] Terlecki-Zaniewicz L, Pils V, Bobbili MR, Lammermann I, Perrotta I, Grillenberger T, et al. Extracellular vesicles in human skin: cross-talk from senescent fibroblasts to keratinocytes by miRNAs. J Invest Dermatol. 2019;139:2425–36. 31220456 10.1016/j.jid.2019.05.015

[107] Rajendran RL, Gangadaran P, Kwack MH, Oh JM, Hong CM, Sung YK, et al. Human fibroblast-derived extracellular vesicles promote hair growth in cultured human hair follicles. FEBS Lett. 2021;595:942–53. 33523480 10.1002/1873-3468.14050

[108] le Riche A, Aberdam E, Marchand L, Frank E, Jahoda C, Petit I, et al. Extracellular vesicles from activated dermal fibroblasts stimulate hair follicle growth through dermal papilla-secreted Norrin. Stem Cells. 2019;37:1166–75. 31237401 10.1002/stem.3043

[109] Moulin V, Castilloux G, Auger FA, Garrel D, O'Connor-McCourt MD, Germain L. Modulated response to cytokines of human wound healing myofibroblasts compared to dermal fibroblasts. Exp Cell Res. 1998;238:283–93. 9457082 10.1006/excr.1997.3827

[110] Barisic-Dujmovic T, Boban I, Clark SH. Fibroblasts/myofibroblasts that participate in cutaneous wound healing are not derived from circulating progenitor cells. J Cell Physiol. 2010;222:703–12. 20020505 10.1002/jcp.21997

[111] Houschyar KS, Borrelli MR, Tapking C, Popp D, Puladi B, Ooms M, et al. Molecular mechanisms of hair growth and regeneration: current understanding and novel paradigms. Dermatology. 2020;236:271–80. 32163945 10.1159/000506155

[112] Matsuzaki T, Yoshizato K. Role of hair papilla cells on induction and regeneration processes of hair follicles. Wound Repair Regen. 1998;6:524–30. 9893172 10.1046/j.1524-475x.1998.60605.x

[113] Chen Y, Huang J, Chen R, Yang L, Wang J, Liu B, et al. Sustained release of dermal papilla-derived extracellular vesicles from injectable microgel promotes hair growth. Theranostics. 2020;10:1454–78. 31938074 10.7150/thno.39566 PMC6956798

[114] Kwack MH, Seo CH, Gangadaran P, Ahn BC, Kim MK, Kim JC, et al. Exosomes derived from human dermal papilla cells promote hair growth in cultured human hair follicles and augment the hair-inductive capacity of cultured dermal papilla spheres. Exp Dermatol. 2019;28:854–7. 30924959 10.1111/exd.13927

[115] Zhou L, Wang H, Jing J, Yu L, Wu X, Lu Z. Regulation of hair follicle development by exosomes derived from dermal papilla cells. Biochem Biophys Res Commun. 2018;500:325–32. 29654758 10.1016/j.bbrc.2018.04.067

[116] Chen Y, Huang J, Liu Z, Chen R, Fu D, Yang L, et al. miR-140-5p in small extracellular vesicles from human papilla cells stimulates hair growth by promoting proliferation of outer root sheath and hair matrix cells. Front Cell Dev Biol. 2020;8:593638. 33425897 10.3389/fcell.2020.593638 PMC7793747

[117] Yan H, Gao Y, Ding Q, Liu J, Li Y, Jin M, et al. Exosomal micro RNAs derived from dermal papilla cells mediate hair follicle stem cell proliferation and differentiation. Int J Biol Sci. 2019;15:1368–82. 31337968 10.7150/ijbs.33233 PMC6643152

[118] Kazi T, Nagata A, Nakagawa T, Matsuzaki T, Inui S. Dermal papilla cell-derived extracellular vesicles increase hair inductive gene expression in adipose stem cells via beta-catenin activation. Cell. 2022;11:202. 10.3390/cells11020202 PMC8773911 35053317

[119] Villarreal-Leal RA, Healey GD, Corradetti B. Biomimetic immunomodulation strategies for effective tissue repair and restoration. Adv Drug Deliv Rev. 2021;179:113913. 34371087 10.1016/j.addr.2021.113913

[120] Wynn TA, Chawla A, Pollard JW. Macrophage biology in development, homeostasis and disease. Nature. 2013;496:445–55. 23619691 10.1038/nature12034 PMC3725458

[121] Englander HR . Fluoridation protects occlusal areas. J Am Dent Assoc. 1979;98:11. 10.14219/jada.archive.1979.0021 282333

[122] Galli SJ, Borregaard N, Wynn TA. Phenotypic and functional plasticity of cells of innate immunity: macrophages, mast cells and neutrophils. Nat Immunol. 2011;12:1035–44. 22012443 10.1038/ni.2109 PMC3412172

[123] Leibovich SJ, Polverini PJ, Shepard HM, Wiseman DM, Shively V, Nuseir N. Macrophage-induced angiogenesis is mediated by tumour necrosis factor-alpha. Nature. 1987;329:630–2. 2443857 10.1038/329630a0

[124] Ploeger DT, Hosper NA, Schipper M, Koerts JA, de Rond S, Bank RA. Cell plasticity in wound healing: paracrine factors of M1/ M2 polarized macrophages influence the phenotypical state of dermal fibroblasts. Cell Commun Signal. 2013;11:29. 23601247 10.1186/1478-811X-11-29 PMC3698164

[125] Suga H, Rennert RC, Rodrigues M, Sorkin M, Glotzbach JP, Januszyk M, et al. Tracking the elusive fibrocyte: identification and characterization of collagen-producing hematopoietic lineage cells during murine wound healing. Stem Cells. 2014;32:1347–60. 24446236 10.1002/stem.1648 PMC4096488

[126] Chen L, Zhang L, Zhang H, Sun X, Liu D, Zhang J, et al. Programmable immune activating electrospun fibers for skin regeneration. Bioact Mater. 2021;6:3218–30. 33778200 10.1016/j.bioactmat.2021.02.022 PMC7966852

[127] Li M, Wang T, Tian H, Wei G, Zhao L, Shi Y. Macrophage-derived exosomes accelerate wound healing through their anti-inflammation effects in a diabetic rat model. Artif Cells Nanomed Biotechnol. 2019;47:3793–803. 31556314 10.1080/21691401.2019.1669617

[128] Gangadaran P, Rajendran RL, Oh JM, Hong CM, Jeong SY, Lee SW, et al. Extracellular vesicles derived from macrophage promote angiogenesis in vitro and accelerate new vasculature formation in vivo. Exp Cell Res. 2020;394:112146. 32561287 10.1016/j.yexcr.2020.112146

[129] Deng F, Yan J, Lu J, Luo M, Xia P, Liu S, et al. M2 macrophage-derived exosomal miR-590-3p attenuates DSS-induced mucosal damage and promotes epithelial repair via the LATS1/YAP/ beta-catenin signalling axis. J Crohns Colitis. 2021;15:665–77. 33075119 10.1093/ecco-jcc/jjaa214

[130] Chen J, Zhou R, Liang Y, Fu X, Wang D, Wang C. Blockade of lncRNA-ASLNCS5088-enriched exosome generation in M2 macrophages by GW4869 dampens the effect of M2 macrophages on orchestrating fibroblast activation. FASEB J. 2019;33:12200–12. 31373848 10.1096/fj.201901610 PMC6902732

[131] Zhu Z, Chen B, Peng L, Gao S, Guo J, Zhu X. Blockade of LINC01605-enriched exosome generation in M2 macrophages impairs M2 macrophage-induced proliferation, migration, and invasion of human dermal fibroblasts. Int J Immunopathol Pharmacol. 2021;35:356479980. 10.1177/20587384211016724 PMC8150463 34011185

[132] Rajendran RL, Gangadaran P, Kwack MH, Oh JM, Hong CM, Gopal A, et al. Engineered extracellular vesicle mimetics from macrophage promotes hair growth in mice and promotes human hair follicle growth. Exp Cell Res. 2021;409:112887. 34678305 10.1016/j.yexcr.2021.112887

[133] Urbich C, Dimmeler S. Endothelial progenitor cells: characterization and role in vascular biology. Circ Res. 2004;95:343–53. 15321944 10.1161/01.RES.0000137877.89448.78

[134] Asahara T, Murohara T, Sullivan A, Silver M, van der Zee R, Li T, et al. Isolation of putative progenitor endothelial cells for angiogenesis. Science. 1997;275:964–7. 9020076 10.1126/science.275.5302.964

[135] Ratajczak J, Kucia M, Mierzejewska K, Marlicz W, Pietrzkowski Z, Wojakowski W, et al. Paracrine proangiopoietic effects of human umbilical cord blood-derived purified CD133+ cells – implications for stem cell therapies in regenerative medicine. Stem Cells Dev. 2013;22:422–30. 23003001 10.1089/scd.2012.0268 PMC3549621

[136] Yong T, Zhang X, Bie N, Zhang H, Zhang X, Li F, et al. Tumor exosome-based nanoparticles are efficient drug carriers for chemotherapy. Nat Commun. 2019;10:3838. 31444335 10.1038/s41467-019-11718-4 PMC6707218

[137] Terriaca S, Fiorelli E, Scioli MG, Fabbri G, Storti G, Cervelli V, et al. Endothelial progenitor cell-derived extracellular vesicles: potential therapeutic application in tissue repair and regeneration. Int J Mol Sci. 2021;22:6375. 10.3390/ijms22126375 PMC8232313 34203627

[138] Xing Z, Zhao C, Liu H, Fan Y. Endothelial progenitor cell-derived extracellular vesicles: a novel candidate for regenerative medicine and disease treatment. Adv Healthc Mater. 2020;9:e2000255. 32378361 10.1002/adhm.202000255

[139] Li X, Chen C, Wei L, Li Q, Niu X, Xu Y, et al. Exosomes derived from endothelial progenitor cells attenuate vascular repair and accelerate reendothelialization by enhancing endothelial function. Cytotherapy. 2016;18:253–62. 26794715 10.1016/j.jcyt.2015.11.009

[140] Li X, Jiang C, Zhao J. Human endothelial progenitor cells-derived exosomes accelerate cutaneous wound healing in diabetic rats by promoting endothelial function. J Diabetes Complicat. 2016;30:986–92. 10.1016/j.jdiacomp.2016.05.009 27236748

[141] Zhang J, Chen C, Hu B, Niu X, Liu X, Zhang G, et al. Exosomes derived from human endothelial progenitor cells accelerate cutaneous wound healing by promoting angiogenesis through Erk1/2 signaling. Int J Biol Sci. 2016;12:1472–87. 27994512 10.7150/ijbs.15514 PMC5166489

[142] Xu J, Bai S, Cao Y, Liu L, Fang Y, Du J, et al. miRNA-221-3p in endothelial progenitor cell-derived exosomes accelerates skin wound healing in diabetic mice. Diabetes Metab Syndr Obes. 2020;13:1259–70. 32368119 10.2147/DMSO.S243549 PMC7183783

[143] Hassanpour M, Cheraghi O, Brazvan B, Hiradfar A, Aghamohammadzadeh N, Rahbarghazi R, et al. Chronic exposure of human endothelial progenitor cells to diabetic condition abolished the regulated kinetics activity of exosomes. Iran J Pharm Res. 2018;17:1068–80. 30127829 PMC6094433

[144] Andrzejewska A, Lukomska B, Janowski M. Concise review: Mesenchymal stem cells: from roots to boost. Stem Cells. 2019;37:855–64. 30977255 10.1002/stem.3016 PMC6658105

[145] Samsonraj RM, Raghunath M, Nurcombe V, Hui JH, van Wijnen AJ, Cool SM. Concise review:Multifaceted characterization of human mesenchymal stem cells for use in regenerative medicine. Stem Cells Transl Med. 2017;6:2173–85. 29076267 10.1002/sctm.17-0129 PMC5702523

[146] Wang M, Xu X, Lei X, Tan J, Xie H. Mesenchymal stem cell-based therapy for burn wound healing. Burns Trauma. 2021;9:b2. 10.1093/burnst/tkab002 PMC8240555 34212055

[147] Sun J, Shi CS, Wang DL. Research advances on the roles of exosomes derived from mesenchymal stem cells in wound healing and prevention and treatment of hypertrophic scars. Zhonghua Shao Shang Za Zhi. 2021;37:495–500. 34044531 10.3760/cma.j.cn501120-20200410-00220 PMC11917257

[148] Zhang W, Hu J, Huang Y, Wu C, Xie H. Urine-derived stem cells: applications in skin, bone and articular cartilage repair. Burns Trauma. 2021;9:b39. 10.1093/burnst/tkab039 PMC8633594 34859109

[149] Zhang B, Wang M, Gong A, Zhang X, Wu X, Zhu Y, et al. HucMSC-exosome mediated-Wnt4 signaling is required for cutaneous wound healing. Stem Cells. 2015;33:2158–68. 24964196 10.1002/stem.1771

[150] Zhang Y, Pan Y, Liu Y, Li X, Tang L, Duan M, et al. Exosomes derived from human umbilical cord blood mesenchymal stem cells stimulate regenerative wound healing via transforming growth factor-beta receptor inhibition. Stem Cell Res Ther. 2021;12:434. 34344478 10.1186/s13287-021-02517-0 PMC8336384

[151] Jiang T, Wang Z, Sun J. Human bone marrow mesenchymal stem cell-derived exosomes stimulate cutaneous wound healing mediates through TGF-beta/Smad signaling pathway. Stem Cell Res Ther. 2020;11:198. 32448395 10.1186/s13287-020-01723-6 PMC7245763

[152] Qian L, Pi L, Fang BR, Meng XX. Adipose mesenchymal stem cell-derived exosomes accelerate skin wound healing via the lncRNA H19/miR-19b/SOX9 axis. Lab Investig. 2021;101:1254–66. 34045678 10.1038/s41374-021-00611-8

[153] Shen K, Wang XJ, Liu KT, Li SH, Li J, Zhang JX, et al. Effects of exosomes from human adipose-derived mesenchymal stem cells on inflammatory response of mouse RAW264.7 cells and wound healing of full-thickness skin defects in mice. Zhonghua Shao Shang Za Zhi. 2022;38:215–26. 35325966 10.3760/cma.j.cn501120-20201116-00477 PMC11705281

[154] Zhang Y, Yan J, Liu Y, Chen Z, Li X, Tang L, et al. Human amniotic fluid stem cell-derived exosomes as a novel cell-free therapy for cutaneous regeneration. Front Cell Dev Biol. 2021;9:685873. 34235150 10.3389/fcell.2021.685873 PMC8255501

[155] Wei P, Zhong C, Yang X, Shu F, Xiao S, Gong T, et al. Exosomes derived from human amniotic epithelial cells accelerate diabetic wound healing via PI3K-AKT-mTOR-mediated promotion in angiogenesis and fibroblast function. Burns Trauma. 2020;8:a20. 10.1093/burnst/tkaa020 PMC7476545 32923490

[156] Su JL, Ma K, Zhang CP, Fu X. Effect of human decidua mesenchymal stem cells-derived exosomes on the function of high glucose-induced senescent human dermal fibroblasts and its possible mechanism. Zhonghua Shao Shang Za Zhi. 2022;38:170–83. 35220706 10.3760/cma.j.cn501120-20210925-00330 PMC11704500

[157] Galkowska H, Wojewodzka U, Olszewski WL. Chemokines, cytokines, and growth factors in keratinocytes and dermal endothelial cells in the margin of chronic diabetic foot ulcers. Wound Repair Regen. 2006;14:558–65. 17014667 10.1111/j.1743-6109.2006.00155.x

[158] Hu ZC, Chen D, Guo D, Liang YY, Zhang J, Zhu JY, et al. Randomized clinical trial of autologous skin cell suspension combined with skin grafting for chronic wounds. Br J Surg. 2015;102:e117–23. 25627123 10.1002/bjs.9688

[159] Xia W, Li M, Jiang X, Huang X, Gu S, Ye J, et al. Young fibroblast-derived exosomal microRNA-125b transfers beneficial effects on aged cutaneous wound healing. J Nanobiotechnology. 2022;20:144. 35305652 10.1186/s12951-022-01348-2 PMC9744129

[160] Ghahary A, Ghaffari A. Role of keratinocyte-fibroblast cross-talk in development of hypertrophic scar. Wound Repair Regen. 2007;15:S46–53. 17727467 10.1111/j.1524-475X.2007.00225.x

[161] Kamerkar S, LeBleu VS, Sugimoto H, Yang S, Ruivo CF, Melo SA, et al. Exosomes facilitate therapeutic targeting of oncogenic KRAS in pancreatic cancer. Nature. 2017;546:498–503. 28607485 10.1038/nature22341 PMC5538883

[162] Barrandon Y, Green H. Three clonal types of keratinocyte with different capacities for multiplication. Proc Natl Acad Sci U S A. 1987;84:2302–6. 2436229 10.1073/pnas.84.8.2302 PMC304638

[163] Yamaguchi Y, Morita A, Maeda A, Hearing VJ. Regulation of skin pigmentation and thickness by Dickkopf 1 (DKK1). J Investig Dermatol Symp Proc. 2009;14:73–5. 10.1038/jidsymp.2009.4 PMC2793095 19675559

[164] Werner S, Krieg T, Smola H. Keratinocyte-fibroblast interactions in wound healing. J Invest Dermatol. 2007;127:998–1008. 17435785 10.1038/sj.jid.5700786

[165] Ackerman JE, Geary MB, Orner CA, Bawany F, Loiselle AE. Obesity/type II diabetes alters macrophage polarization resulting in a fibrotic tendon healing response. PLoS One. 2017;12:e181127. 10.1371/journal.pone.0181127 PMC5501654 28686669

[166] Patel S, Srivastava S, Singh MR, Singh D. Mechanistic insight into diabetic wounds: pathogenesis, molecular targets and treatment strategies to pace wound healing. Biomed Pharmacother. 2019;112:108615. 30784919 10.1016/j.biopha.2019.108615

[167] Huang P, Bi J, Owen GR, Chen W, Rokka A, Koivisto L, et al. Keratinocyte microvesicles regulate the expression of multiple genes in dermal fibroblasts. J Invest Dermatol. 2015;135:3051–9. 26288358 10.1038/jid.2015.320

[168] Cheung KL, Jarrett R, Subramaniam S, Salimi M, Gutowska-Owsiak D, Chen YL, et al. Psoriatic T cells recognize neolipid antigens generated by mast cell phospholipase delivered by exosomes and presented by CD1a. J Exp Med. 2016;213:2399–412. 27670592 10.1084/jem.20160258 PMC5068234

[169] Shao S, Fang H, Zhang J, Jiang M, Xue K, Ma J, et al. Neutrophil exosomes enhance the skin autoinflammation in generalized pustular psoriasis via activating keratinocytes. FASEB J. 2019;33:6813–28. 30811955 10.1096/fj.201802090RR

[170] Kim M, Park Y, Kwon Y, Kim Y, Byun J, Jeong MS, et al. MiR-135-5p-p62 axis regulates autophagic flux, tumorigenic potential, and cellular interactions mediated by extracellular vesicles during allergic inflammation. Front Immunol. 2019;10:738. 31024564 10.3389/fimmu.2019.00738 PMC6460569

[171] Kim M, Jo H, Kwon Y, Jeong MS, Jung HS, Kim Y, et al. MiR-154-5p-MCP1 axis regulates allergic inflammation by mediating cellular interactions. Front Immunol. 2021;12:663726. 34135893 10.3389/fimmu.2021.663726 PMC8201518

[172] Freund A, Orjalo AV, Desprez PY, Campisi J. Inflammatory networks during cellular senescence: causes and consequences. Trends Mol Med. 2010;16:238–46. 20444648 10.1016/j.molmed.2010.03.003 PMC2879478

[173] Dimri GP, Lee X, Basile G, Acosta M, Scott G, Roskelley C, et al. A biomarker that identifies senescent human cells in culture and in aging skin in vivo. Proc Natl Acad Sci U S A. 1995;92:9363–7. 7568133 10.1073/pnas.92.20.9363 PMC40985

[174] Campisi J . Aging, cellular senescence, and cancer. Annu Rev Physiol. 2013;75:685–705. 23140366 10.1146/annurev-physiol-030212-183653 PMC4166529

[175] Demaria M, Desprez PY, Campisi J, Velarde MC. Cell autonomous and non-autonomous effects of senescent cells in the skin. J Invest Dermatol. 2015;135:1722–6. 25855157 10.1038/jid.2015.108 PMC4466004

[176] Yin Y, Chen H, Wang Y, Zhang L, Wang X. Roles of extracellular vesicles in the aging microenvironment and age-related diseases. J Extracell Vesicles. 2021;10:e12154. 34609061 10.1002/jev2.12154 PMC8491204

[177] Gao J, Wang S, Wang Z. High yield, scalable and remotely drug-loaded neutrophil-derived extracellular vesicles (EVs) for anti-inflammation therapy. Biomaterials. 2017;135:62–73. 28494264 10.1016/j.biomaterials.2017.05.003 PMC5516786

[178] Taylor CJ, Bolton EM, Bradley JA. Immunological considerations for embryonic and induced pluripotent stem cell banking. Philos Trans R Soc Lond Ser B Biol Sci. 2011;366:2312–22. 21727137 10.1098/rstb.2011.0030 PMC3130422

[179] Zhang W, Bai X, Zhao B, Li Y, Zhang Y, Li Z, et al. Cell-free therapy based on adipose tissue stem cell-derived exosomes promotes wound healing via the PI3K/Akt signaling pathway. Exp Cell Res. 2018;370:333–42. 29964051 10.1016/j.yexcr.2018.06.035

[180] Jeppesen DK, Fenix AM, Franklin JL, Higginbotham JN, Zhang Q, Zimmerman LJ, et al. Reassessment of exosome composition. Cell. 2019;177:428–45. 30951670 10.1016/j.cell.2019.02.029 PMC6664447

[181] Ramirez MI, Amorim MG, Gadelha C, Milic I, Welsh JA, Freitas VM, et al. Technical challenges of working with extracellular vesicles. Nanoscale. 2018;10:881–906. 29265147 10.1039/c7nr08360b

[182] Royo F, Thery C, Falcon-Perez JM, Nieuwland R, Witwer KW. Methods for separation and characterization of extracellular vesicles: results of a worldwide survey performed by the ISEV Rigor and Standardization subcommittee. Cell. 2020;9:1955. 10.3390/cells9091955 PMC7563174 32854228

[183] Wang C, Wang M, Xu T, Zhang X, Lin C, Gao W, et al. Engineering bioactive self-healing antibacterial exosomes hydrogel for promoting chronic diabetic wound healing and complete skin regeneration. Theranostics. 2019;9:65–76. 30662554 10.7150/thno.29766 PMC6332800

[184] Shen Y, Xu G, Huang H, Wang K, Wang H, Lang M, et al. Sequential release of small extracellular vesicles from bilayered thiolated alginate/polyethylene glycol diacrylate hydrogels for scarless wound healing. ACS Nano. 2021;15:6352–68. 33723994 10.1021/acsnano.0c07714

[185] Da SL, Reis RL, Correlo VM, Marques AP. Hydrogel-based strategies to advance therapies for chronic skin wounds. Annu Rev Biomed Eng. 2019;21:145–69. 30822099 10.1146/annurev-bioeng-060418-052422

[186] Qian Z, Bai Y, Zhou J, Li L, Na J, Fan Y, et al. A moisturizing chitosan-silk fibroin dressing with silver nanoparticles-adsorbed exosomes for repairing infected wounds. J Mater Chem B. 2020;8:7197–212. 32633312 10.1039/d0tb01100b

[187] Shiekh PA, Singh A, Kumar A. Exosome laden oxygen releasing antioxidant and antibacterial cryogel wound dressing OxOBand alleviate diabetic and infectious wound healing. Biomaterials. 2020;249:120020. 32305816 10.1016/j.biomaterials.2020.120020

[188] Su D, Tsai HI, Xu Z, Yan F, Wu Y, Xiao Y, et al. Exosomal PD-L1 functions as an immunosuppressant to promote wound healing. J Extracell Vesicles. 2019;9:1709262. 33133428 10.1080/20013078.2019.1709262 PMC7580831

[189] Li X, Wang Y, Shi L, Li B, Li J, Wei Z, et al. Magnetic targeting enhances the cutaneous wound healing effects of human mesenchymal stem cell-derived iron oxide exosomes. J Nanobiotechnology. 2020;18:113. 32799868 10.1186/s12951-020-00670-x PMC7429707

[190] Kalluri R, LeBleu VS. The biology, function, and biomedical applications of exosomes. Science. 2020;367:eaau6977. 10.1126/science.aau6977 PMC7717626 32029601

[191] Charoenviriyakul C, Takahashi Y, Morishita M, Matsumoto A, Nishikawa M, Takakura Y. Cell type-specific and common characteristics of exosomes derived from mouse cell lines: yield, physicochemical properties, and pharmacokinetics. Eur J Pharm Sci. 2017;96:316–22. 27720897 10.1016/j.ejps.2016.10.009

[192] Venugopal C, Shamir C, Senthilkumar S, Babu JV, Sonu PK, Nishtha KJ, et al. Dosage and passage dependent neuroprotective effects of exosomes derived from rat bone marrow mesenchymal stem cells: an in vitro analysis. Curr Gene Ther. 2017;17:379–90. 29366415 10.2174/1566523218666180125091952

[193] Skuratovskaia D, Vulf M, Khaziakhmatova O, Malashchenko V, Komar A, Shunkin E, et al. Exosome limitations in the treatment of inflammatory diseases. Curr Pharm Des. 2021;27:3105–21. 33302851 10.2174/1381612826666201210120444

[194] Kwon HH, Yang SH, Lee J, Park BC, Park KY, Jung JY, et al. Combination treatment with human adipose tissue stem cell-derived exosomes and fractional CO2 laser for acne scars: a 12-week prospective, double-blind, randomized split-face study. Acta Derm Venereol. 2020;100:adv00310. 33073298 10.2340/00015555-3666 PMC9309822

